# Lnc-ITSN1-2, Derived From RNA Sequencing, Correlates With Increased Disease Risk, Activity and Promotes CD4^+^ T Cell Activation, Proliferation and Th1/Th17 Cell Differentiation by Serving as a ceRNA for IL-23R via Sponging miR-125a in Inflammatory Bowel Disease

**DOI:** 10.3389/fimmu.2020.00852

**Published:** 2020-05-28

**Authors:** Jiayan Nie, Qiu Zhao

**Affiliations:** ^1^Department of Gastroenterology, Zhongnan Hospital of Wuhan University, Wuhan, China; ^2^The Hubei Clinical Center & Key Laboratory of Intestinal & Colorectal Diseases, Wuhan, China

**Keywords:** inflammatory bowel disease, lncRNA profiles, lnc-ITSN1-2, disease risk, disease activity, CD4^+^ T cell

## Abstract

**Background:** This study aimed to investigate long-non-coding RNA (lncRNA) expression profiles and the correlation of lnc-ITSN1-2 expression with disease risk, activity and inflammation, and its influence on CD4^+^ T cell activation, proliferation, and differentiation of inflammatory bowel disease (IBD).

**Methods:** LncRNA expression profiles were detected in intestinal mucosa samples from six IBD patients and six healthy controls (HCs). Intestinal mucosa and PBMC lnc-ITSN1-2, IL-23R, and inflammatory cytokines were measured in 120 IBD patients [60 Crohn's disease (CD) and 60 ulcerative colitis (UC)] and 30 HCs. Effect of lnc-ITSN1-2 on IBD CD4^+^ T cell activation, proliferation, and differentiation was determined and its regulatory interaction with miR-125a and IL-23R was detected.

**Results:** Three-hundred-and-nine upregulated and 310 downregulated lncRNAs were identified in IBD patients by RNA-Sequencing, which were enriched in regulating immune and inflammation related pathways. Large-sample qPCR validation disclosed that both intestinal mucosa and PBMC lnc-ITSN1-2 expressions were increased in IBD patients compared to HCs, and presented with good predictive values for IBD risk, especially for active disease conditions, and they positively correlated with disease activity, inflammation cytokines, and IL-23R in IBD patients. Lnc-ITSN1-2 was decreased after infliximab treatment in active-CD patients. Furthermore, lnc-ITSN1-2 promoted IBD CD4^+^ T cell activation and proliferation, and stimulated Th1/Th17 cell differentiation. Multiple rescue experiments disclosed that lnc-ITSN1-2 functioned in IBD CD4^+^ T cells via targeting miR-125a, then positively regulating IL-23R. Luciferase Reporter assay observed that lnc-ITSN1-2 bound miR-125a, and miR-125a bound IL-23R.

**Conclusion:** Lnc-ITSN1-2 correlates with increased disease risk, activity, and inflammatory cytokines of IBD, and promotes IBD CD4^+^ T cell activation, proliferation, and Th1/Th17 cell differentiation by serving as a competing endogenous RNA for IL-23R via sponging miR-125a.

## Introduction

Inflammatory bowel disease (IBD), mainly consisting of Crohn's disease (CD) and ulcerative colitis (UC), is a severe gastrointestinal disorder that affects people of all ages worldwide with a continuously increasing incidence (1, 2). Generally, IBD is considered to result from aberrant and continuing immune responses to the microbes in the gut, and is featured by the genetic susceptibility of individuals (3). Despite the fact that the exact pathogenesis of IBD is still largely unknown, it involves a complex interaction between genetics, environmental factors, and immune responses, among which genetic studies have recently made the most rapid progress (1–3).

Long non-coding RNA (lncRNA) is a class of RNAs with a length over 200 nucleotides and presents with few protein-coding functions, which have been reported to participate in many pathological processes via epigenetic regulation, gene imprinting, transcriptional activation, mRNA modification, nuclear transportation, and protein viability activation in various diseases (4–7). Specifically, several studies have disclosed that lncRNAs are greatly implicated in the pathogenesis of inflammatory diseases such as rheumatoid arthritis (RA), osteoarthritis (OA), and systemic lupus erythematosus (SLE) (8–10). As for IBD, limited data about the role of lncRNAs are reported and only a limited number of lncRNAs have been discovered to be involved in its pathogenesis (11, 12). A previous study reveals that lncRNA ANRIL improves UC development via regulating the miR-323b-5p/TLR4/MyD88/NF-κB pathway (13). Another study illustrates that lncRNA DQ786243 is upregulated in active CD patients compared to remission CD patients and healthy controls (HCs), and it increases CREB and Foxp3 expression (11). Furthermore, 15 lncRNAs are observed to be differentially expressed in two mouse models of colitis by RNA-sequencing (14). However, the functions of most lncRNAs in IBD development and progression are still vastly unknown, and is in great need of further investigation.

In this present study, we explored the intestinal mucosa lncRNA expression profile by RNA sequencing in IBD patients and HCs and observed that lncRNA ITSN1-2 (lnc-ITSN1-2) was one of the most dysregulated genes. Additionally, lnc-ITSN1-2 is reported to be implicated in the pathology of RA (15) and may serve as a potential biomarker for coronary artery disease risk (16), thus we hypothesized that lnc-ITSN1-2 might be involved in IBD pathogenesis as well. Then we aimed to investigate the correlation of intestinal mucosa and PBMC lnc-ITSN1-2 expressions with disease risk, activity, and inflammation of IBD, and to further explore the effect of lnc-ITSN1-2 on regulating IBD CD4^+^ T cell activation, proliferation, and differentiation as well as its potential molecular mechanism.

## Materials and Methods

### Participants

One-hundred-and-twenty IBD patients [including 30 active CD (A-CD) patients, 30 CD with remission (R-CD) patients, 30 active UC (A-UC) patients, 30 UC with remission (R-UC) patients], and 30 HCs from Zhongnan Hospital of Wuhan University were recruited in this study from November 2015 to October 2017. All IBD patients were diagnosed according to clinical characteristics, radiological findings, endoscopic examination, and histological results, and were aged above 18 years. The healthy volunteers who underwent endoscopy, with age and gender matched to total IBD patients, served as HCs. This study was approved by the Ethics Committee of Zhongnan Hospital of Wuhan University and was conducted in accordance with the Declaration of Helsinki. All participants provided written informed consent.

### Data Collection, Definition, and Sample Obtaining

Age, gender, C-reactive protein (CRP), and erythrocyte sedimentation rate (ESR) of all participants were collected. Additionally, Crohn's disease activity index (CDAI) for CD patients and Mayo index for UC patients were calculated to assess the clinical disease severity. A-CD was defined as a CDAI score above 150 points; A-UC was defined as Mayo above 2 points. Intestinal mucosa samples (lesion site for IBD patients and normal tissue for HCs) were obtained during the endoscopy and peripheral blood mononuclear cell (PBMC) samples were obtained through centrifugation of peripheral blood from all the participants.

### RNA Sequencing

Intestinal mucosa samples of six active IBD patients (including three A-CD and three A-UC patients) and six age and gender matched HCs from the total participants were used for RNA sequencing to detect lncRNA and mRNA expression profiles. In brief, after the extraction of total RNA by Trizol reagent (Invitrogen, USA), a concentration, purity, and integrity quality control was performed. Ribosomal RNA was then removed from RNA using the Epicenter Ribo-zero™ rRNA Removal Kit (Epicenter, USA), and the remaining RNA was used for the sequencing library. Subsequently, the first and second strands of the cDNA were synthesized and library fragments were purified using the AMPure XP system to select cDNA fragments with a length of 150–200 bp. A polymerase chain reaction (PCR) assay was then conducted and quality of the library was measured by the Bioanalyzer 2100 system. Clustering of index-coded samples was then performed using the HiSeq PE Cluster Kit v4 cBot (Illumina, USA). The library was sequenced on the Illumina Hiseq X10 platform and 150 bp paired-end reads were produced after cluster generation. Automate quality control and adapter trimming were conducted using Trim Galore, Cutadapt, and FastQC. The trimmed reads were then mapped to the human genome Hg38 by HISAT2 with the default parameters, and mapping quality control was conducted using RSeQC. The reads count of lncRNA and mRNA were then calculated using the FeatureCounts package based on the annotation file (Homo_sapiens.GRCh38.83.gtf) in the Ensembl database.

### Bioinformatics

Bioinformatics analyses were performed using R software (Version 3.3.3). In brief, (1) Genes which were discovered in 50% or more samples were selected for bioinformatics analysis, then the raw reads counts were normalized, and logarithmic transformation was applied. The differentially expressed lncRNAs (DELs) were detected using DeSeq2, the statistical significance was defined as adjusted P (*P*_adj_) value <0.05, and the biological significance was defined as a difference of at least 2.0-folds, that is abs [log2 (fold change)]>1.0. Subsequently, Valcano Plots were drawn to present DELs. (2) Heatmap plots of DELs were completed by pheatmap package. (3) Gene Ontology (GO) and Kyoko Encyclopedia of Genes and Genomes (KEGG) enrichment analyses of DELs were performed using DAVID web servers based on the regulated mRNA profiles. (4) A regulatory network of the top 10 upregulated DELs and 10 downregulated DELs was drawn by igraph package.

### Measurement of lnc-ITSN1-2, IL-23R, and Inflammatory Cytokines in Intestinal Mucosa and PBMC Samples

Since lnc-ITSN1-2 was one of the most dysregulated DELs in IBD patients in our RNA sequencing data, and because it was reported to be implicated in the pathology of RA (15), we further investigated the mechanism of lnc-ITSN1-2 dysregulation in IBD. Lnc-ITSN1-2 expression in both intestinal mucosa and PBMC samples from a total of 120 IBD patients and 30 HCs was measured using real-time quantitative polymerase chain reaction (RT-qPCR), and its potential target gene IL-23R expression was also detected. Additionally, five inflammatory cytokine (including INF-γ, TNF-α, IL-6, IL-10, IL-17, IL-1β, IL-18) expressions in both intestinal mucosa and PBMC samples, from a total of 120 IBD patients, were measured using RT-qPCR as well.

### Infliximab (IFX) Treatment for A-CD Patients

Among 30 A-CD patients, 21 cases initiated IFX (Xian Janssen, China) treatment (5 mg/kg at week 0, 2, and 6) according to the manufacturer's instructions. Intestinal mucosa and PBMC samples were collected at week 0 and 12, and lnc-ITSN1-2 expression in samples was detected by RT-qPCR. The clinical efficacy of IFX was assessed at week 12 after initial infusion of IFX in this study, and these patients were categorized into two groups (response and non-response) according to changes in the CDAI scores. In brief, patients with a decrease in the CDAI score of ≥70 points after IFX treatment were placed in the response group, while patients with a change in the CDAI score of <70 points were placed into the failure group (17).

### Isolation of Peripheral Blood CD4^+^ T Cells From A-CD Patients, A-UC Patients, and HCs

CD4^+^ T cells were isolated from 10 A-CD patients, 10 A-UC patients, and 10 HCs according to the method described in our previous study (18). In brief, PBMC samples were collected and isolated using Anti-Human CD4 Particles (BD, USA), and the purity of CD4^+^ T cells was detected using flow cytometry (BD, USA) (purity was acquired to be above 95%).

### Transfection

Green-fluorescent protein carried the lentivirus encoding empty vector, lnc-ITSN1-2 overexpression, and lnc-ITSN1-2 shRNA—constructed by Shanghai Gene Company (Shanghai, China), and were transfected into CD4^+^ T cells as the LV-scramble group, LV-lnc-ITSN1-2 group, and LV-anti-lnc-ITSN1-2 group according to the method described in our previous study (18). CD4^+^ T cells without lentivirus transfection were used as the blank control (Blank group) in this study. For transfection, in brief, CD4^+^ T cells were stimulated by 5 mg/mL anti-human-CD3 (Abcam, USA), and 2 mg/mL anti-human-CD28 (Abcam, USA) for 24 h, then lentivirus with 100 multiplicity of infection (MOI) was incubated with CD4^+^T cells overnight. Subsequently, transfected cells were cultured with 5 mg/mL anti-human-CD3 (Abcam, USA) and 2mg/mL anti-human-CD28 (Abcam, USA) for another 72 h.

### CD4^+^ T Cell Activation

Transfected CD4^+^ T cells of each group were cultured for 72 h and then stained by anti-human-CD25 and anti-human-CD69, and the percentages of CD25^+^ cells and CD69^+^ cells were analyzed using flow cytometry (BD, USA), to measure CD4^+^ T cell activation.

### CD4^+^ T Cell Proliferation

Transfected CD4^+^ T cells of each group were cultured, and then cell proliferation at 0, 24, 48, and 72 h was detected using a CCK8 assay. In brief, after culturing, cells were centrifuged and the supernatant was discarded, then 10 ul Cell Counting Kit-8 solution (Sigma, USA) and serum free medium was added in the plate and cultured for 2 h. Then the OD value of 450 nm was detected using the Microplate Reader (Thermo, USA).

### Th1/Th17 Cell Differentiation

Transfected CD4^+^ T cells of each group were cultured for 72 h, and then IFN-γ, TNF-α, IL-17, T-bet, and RORC mRNA expressions were detected using RT-qPCR assay to detect the Th1/Th17 cell differentiation.

### Rescue Experiment: IL-23R Overexpression in lnc-ITSN1-2 Knockdown Treated CD4^+^ T Cells

Lentivirus encoding lnc-ITSN1-2 shRNA, and encoding lnc-ITSN1-2 shRNA/IL-23R overexpression were constructed by Shanghai Gene Company (Shanghai, China), and were transfected into CD4^+^ T cells as the LV-anti-lnc-ITSN1-2 group and LV-anti-lnc-ITSN1-2 & LV-IL-23R group. Lnc-ITSN1-2 expression, IL-23R mRNA expression, CD4^+^ T cell activation, CD4^+^ T cell proliferation, and Th1/Th17 differentiation was detected according to the methods described above.

### Rescue Experiment: miR-125a Knockdown in lnc-ITSN1-2 Knockdown Treated CD4^+^ T Cells

Lentivirus encoding lnc-ITSN1-2 shRNA, and encoding lnc-ITSN1-2 shRNA/miR-125a shRNA were constructed by Shanghai Gene Company (Shanghai, China), and were transfected into CD4^+^ T cells as the LV-anti-lnc-ITSN1-2 group and LV-anti-lnc-ITSN1-2 & LV-anti-miR-125a group. Lnc-ITSN1-2 expression, miR-125a expression, CD4^+^ T cell activation, CD4^+^ T cell proliferation, and Th1/Th17 differentiation was detected according to the methods described above.

### Rescue Experiment: IL-23R Knockdown in miR-125a Knockdown Treated CD4^+^ T Cells

Lentivirus encoding miR-125a shRNA, and encoding miR-125a shRNA/IL-23R shRNA were constructed by Shanghai Gene Company (Shanghai, China), and were transfected into CD4^+^ T cells as the LV-anti-miR-125a group and LV-anti-miR-125a & LV-anti-IL23R group. MiR-125a expression, IL-23R mRNA expression, CD4^+^ T cell activation, CD4^+^ T cell proliferation, and Th1/Th17 differentiation was detected according to the methods described above.

### Luciferase Reporter Assay

In order to confirm the binding between lnc-ITSN1-2 and miR-125a, between miR-125a and IL-23R, a Luciferase Reporter assay was performed. In brief, 293T cells were purchased from American Typical Culture Collection (Virginia, USA). Mutant sequences of the binding site were designed and according vectors were constructed by Shanghai Gene Company (Shanghai, China). The transfection and detections were performed according to a previous study (19).

### JAK2/STAT3 Pathway and NF-κB Pathway Measurement

Lentivirus encoding empty vector, encoding lnc-ITSN1-2 shRNA, and encoding lnc-ITSN1-2 shRNA/IL-23R overexpression were constructed by Shanghai Gene Company (Shanghai, China), and were transfected into IBD CD4^+^ T cells as the LV-scramble group, LV-anti-lnc-ITSN1-2 group, and LV-anti-lnc-ITSN1-2 & LV-IL-23R group. After 72 h transfection, JAK2, pJAK2, STAT3, pSTAT3, IKKA, and NF-κB expressions were detected by Western Blot assay according to the methods described in our previous study (16). The antibodies are listed in Supplementary Table 1.

### RT-qPCR

Total RNA was extracted from tissues or cells using Trizol reagent (Invitrogen, USA), and reversely transcribed into cDNA using the 5 × All-In-One RT MasterMix kit (Applied Biological Materials, Canada). The RT-qPCR was performed using a SYPR Green PCR kit (TaKaRa, Japan). LncRNA and mRNA expressions were calculated using the 2-^ΔΔ*Ct*^ method, and GAPDH was used as the internal reference. The detailed primers used in this study are listed in Supplementary Table 2.

### Statistics

Bioinformatics analysis of RNA sequencing was performed using R software (Version 3.3.3), and the detailed analysis methods are presented in the aforementioned “Bioinformatics” Subsection. Statistics were performed using SPSS 21.0 Software (IBM, USA) and graphs were made using GraphPad Prism 6.01 Software (GraphPad Int., USA). Comparisons among groups were determined by a Kruskal-Wallis H rank sum test followed by a two-group Wilcoxon rank sum test or One-way ANOVA test, followed by a multiple comparison test. Comparisons between the two groups were determined by a *t*-test or Wilcoxon rank sum test. Comparison between the two time points in one group was determined by the Wilcoxon signed rank sum test. Correlation was determined by the Spearman test. The receiver operating characteristic (ROC) curve was drawn, and area under curve (AUC) was calculated to assess the ability of a specific index to distinguish two subjects. A *P* < 0.05 was considered as significant.

## Results

### Analysis of lncRNA Expression Profiles in IBD

**Figure 1:**
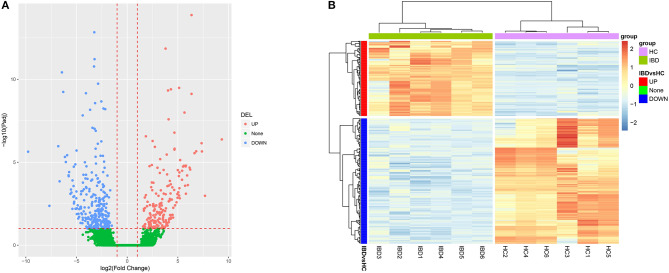
Bioinformatics analysis of RNA sequencing. **(A)** Volcano Plot; **(B)** Heatmap analysis for DELs. DELs, differentially expressed lncRNAs.

Three-hundred-and-nine upregulated lncRNAs and 310 downregulated lncRNAs were identified in intestinal mucosa samples from six IBD patients compared to six HCs by Valcano Plot (Figure 1A). Heatmap analysis revealed that these DELs distinguished IBD patients from HCs well (Figure 1B). GO enrichment analysis disclosed that DELs were enriched in the molecular function (such as structural constituent of muscle, actin binding, and CXCR3 chemokine receptor binding), the cellular component (such as the integral component of the plasma membrane, the external side of the plasma membrane and Z disc), and the biological process (such as muscle filament sliding, inflammatory response, and muscle contraction) (Supplementary Figure 1A). KEGG enrichment analysis illuminated that DELs were enriched in the regulation of primary immunodeficiency, cytokine-cytokine receptor interaction, cAMP signaling pathways and so on (Supplementary Figure 1B). In addition, the top 10 upregulated and the top 10 downregulated DELs in IBD patients compared to HCs were selected by rank of absolute value of Log_2_FC which are listed in Table 1, and the regulatory network of these 20 DELs was analyzed and is shown in Supplementary Figure 2. Collectively, these data indicated that the lncRNA expression profile plays a critical role in IBD pathogenesis via regulating multiple immune and inflammation related pathways.

**Table 1:** Top 10 upregulated and 10 downregulated DELs in IBD patients compared to HCs.

**Gene ID**	**Gene symbol**	**Chromosome**	**Log_**2**_FC**	***P***	***P*_**adj**_**	**Trend**
ENSG00000237111	IGHJ3P	chr14	9.344263	4.7E-09	4.22E-07	UP
ENSG00000253998	IGKV2-29	chr2	7.678376	3.5E-05	0.001048	UP
ENSG00000237945	lnc-ITSN1-2	chr21	7.378726	3.06E-08	2.21E-06	UP
ENSG00000253701	AL928768.3	chr14	7.341898	8.52E-09	7.1E-07	UP
ENSG00000224039	AC091493.1	chr3	6.814635	3E-08	2.18E-06	UP
ENSG00000228651	RP11-556E13.1	chr10	6.64548	3.92E-08	2.8E-06	UP
ENSG00000230838	LINC01614	chr2	6.354923	4.38E-17	1.32E-14	UP
ENSG00000226777	KIAA0125	chr14	6.35255	4.71E-12	7.45E-10	UP
ENSG00000265714	AC246787.4	chr14	6.228509	5.31E-07	2.74E-05	UP
ENSG00000264781	MIR4537	chr14	6.162295	3.85E-07	2.08E-05	UP
ENSG00000174407	MIR1-1HG	chr20	−9.77148	3.19E-08	2.29E-06	DOWN
ENSG00000265142	MIR133A1HG	chr18	−7.67565	0.000176	0.004265	DOWN
ENSG00000261244	RP11-114H24.7	chr15	−6.80165	1.34E-08	1.08E-06	DOWN
ENSG00000271199	RP13-216E22.5	chrX	−6.6696	3.5E-06	0.000142	DOWN
ENSG00000239776	AC079949.1	chr12	−6.44043	1.88E-13	3.78E-11	DOWN
ENSG00000241781	AL161626.1	chr9	−6.30676	3.51E-12	5.72E-10	DOWN
ENSG00000231335	AC107072.2	chr4	−6.14081	7.25E-08	4.77E-06	DOWN
ENSG00000119440	LCN1P1	chr9	−6.0525	1.57E-07	9.57E-06	DOWN
ENSG00000226306	NPY6R	chr5	−5.93067	5.48E-08	3.73E-06	DOWN
ENSG00000228725	MTND2P12	chr17	−5.78065	1.35E-06	6.12E-05	DOWN

*Top 10 upregulated and 10 downregulated DELs in IBD patients compared to HCs were selected by rank of absolute value of Log_2_FC. DEL, differentially expressed lncRNA; IBD, inflammatory bowel disease; HC, health control; ID, identification; FC, fold change; P_adj_, adjusted P*.

### Lnc-ITSN1-2 Expression in IBD Patients and HCs

**Figure 2:**
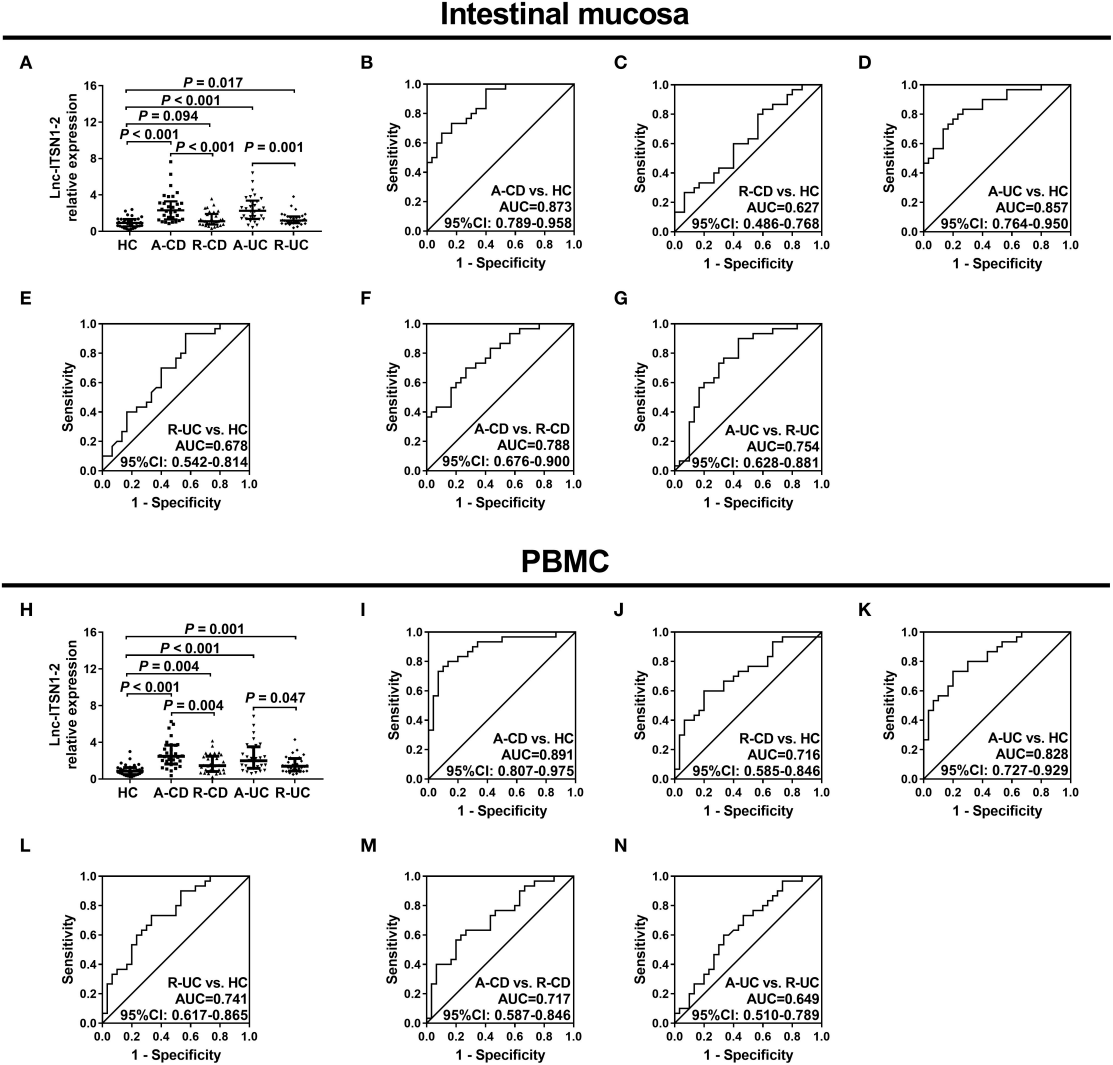
Correlation of lnc-ITSN1-2 with IBD risk. **(A)** Comparison of intestinal mucosa lnc-ITSN1-2 among HC, A-CD, R-CD, A-UC, and R-UC; **(B)** ROC curve of intestinal mucosa lnc-ITSN1-2 (A-CD vs. HC); **(C)** ROC curve of intestinal mucosa lnc-ITSN1-2 (R-CD vs. HC); **(D)** ROC curve of intestinal mucosa lnc-ITSN1-2 (A-UC vs. HC); **(E)** ROC curve of intestinal mucosa lnc-ITSN1-2 (R-UC vs. HC); **(F)** ROC curve of intestinal mucosa lnc-ITSN1-2 (A-CD vs. R-CD); **(G)** ROC curve of intestinal mucosa lnc-ITSN1-2 (A-UC vs. R-UC); **(H)** Comparison of PBMC lnc-ITSN1-2 among HC, A-CD, R-CD, A-UC, and R-UC; **(I)** ROC curve of PBMC lnc-ITSN1-2 (A-CD vs. HC); **(J)** ROC curve of PBMC lnc-ITSN1-2 (R-CD vs. HC); **(K)** ROC curve of PBMC lnc-ITSN1-2 (A-UC vs. HC); **(L)** ROC curve of PBMC lnc-ITSN1-2 (R-UC vs. HC); **(M)** ROC curve of PBMC lnc-ITSN1-2 (A-CD vs. R-CD); **(N)** ROC curve of PBMC lnc-ITSN1-2 (A-UC vs. R-UC). IBD, inflammatory bowel disease; HC, health control; A-CD, active Crohn's disease; R-CD, Crohn's disease with remission; A-UC, active ulcerative colitis; R-UC, ulcerative colitis with remission; ROC, receiver operator characteristic; PBMC, peripheral blood mononuclear cells.

Since lnc-ITSN1-2 was one of the most dysregulated DELs in IBD patients, as indicated by our RNA sequencing data (Table 1), and it was reported to be implicated in the pathology of RA (15) and might serve as a potential biomarker for coronary artery disease risk (16). We therefore hypothesized that lnc-ITSN1-2 might be involved in IBD development and progression. Thus, we further validated lnc-ITSN1-2 expression in both intestinal mucosa and PBMC samples from 120 IBD (including 30 A-CD, 30 R-CD, 30 A-UC and 30 R-UC) patients and 30 HCs using RT-qPCR. Age or gender was of no difference among 30 A-CD, 30 R-CD, 30 A-UC, 30 R-UC, and 30 HCs, and other clinical characteristics of IBD patients and HCs are shown in Table 2. We observed that intestinal mucosa lnc-ITSN1-2 was upregulated in A-CD patients, A-UC patients, and R-UC patients compared to HCs, while only numerically elevated in R-CD patients compared to HCs (but without statistical significance) (Figure 2A). ROC curves analysis further disclosed that intestinal mucosa lnc-ITSN1-2 presented with an excellent value in predicting A-CD risk with AUC 0.873 (95% CI 0.789–0.958) (Figure 2B) and A-UC risk with AUC 0.857 (95% CI 0.764–0.950) (Figure 2D), and a mild value in predicting R-UC risk with AUC 0.678 (95% CI 0.542–0.814) (Figure 2E), while it had no value in predicting R-CD risk with AUC 0.627 (95% CI 0.486–0.768) (Figure 2C), compared to HC. As to PBMC lnc-ITSN1-2, it was discovered to be upregulated in A-CD patients, R-CD patients, A-UC patients, and R-UC patients compared to HCs (Figure 2H). ROC curves analysis further exhibited that PBMC lnc-ITSN1-2 illuminated an excellent value in predicting A-CD risk with AUC 0.891 (95% CI 0.807-0.975) (Figure 2I) and A-UC risk with AUC 0.828 (95% CI 0.727–0.929) (Figure 2K), and a mild value in predicting R-CD risk with AUC 0.716 (95% CI 0.585–0.846) (Figure 2J) and R-UC risk with AUC 0.741 (95% CI 0.617–0.865) (Figure 2L). Collectively, these data implied that lnc-ITSN1-2 possessed potential to be a biomarker of IBD development and it might be implicated in the pathology of IBD. Furthermore, we also observed that intestinal mucosa lnc-ITSN1-2 was increased in A-CD patients compared to R-CD patients, and elevated in A-UC patients compared to R-UC patients, meanwhile, ROC curve analyses showed that intestinal mucosa lnc-ITSN1-2 presented with an acceptable value in distinguishing A-CD patients from R-CD patients, and A-UC patients from R-UC patients (Figures 2A,F,G). PBMC lnc-ITSN1-2 was increased in A-CD patients compared to R-CD patients, and was elevated in A-UC patients compared to R-UC patients, furthermore, ROC curve analyses showed that wPBMC lnc-ITSN1-2 presented with some value in distinguishing A-CD patients from R-CD patients, and A-UC patients from R-UC patients (Figures 2H,M,N), but the differentiating ability was weaker than intestinal mucosa lnc-ITSN1-2 to some extent. These data uncovered the potential of lnc-ITSN1-2 as a biomarker for the active disease of IBD (CD and UC) as well.

**Table 2:** The clinical characteristics of IBD patients and HCs.

**Characteristics**	**HC (*N* = 30)**	**A-CD (*N* = 30)**	**R-CD (*N* = 30)**	**A-UC (*N* = 30)**	**R-UC (*N* =3 0)**	***P*-value**
Age (years)	36.7 (10.7)	34.3 (9.6)	36.0 (9.8)	39.9 (12.1)	37.9 (11.3)	0.349
Male/female	14/16	17/13	11/19	16/14	14/16	0.586
CRP (mg/L)	2.9 (2.0–4.6)	52.4 (36.1–68.9)	17.4 (12.8–28.3)	49.3 (37.9–77.0)	19.8 (15.7–25/0)	<0.001
ESR (mm/h)	9.9 (7.9–13.4)	47.5 (39.3–61.8)	19.5 (13.1–29.3)	44.3 (35.3–54.5)	19.1 (12.7–22.3)	<0.001
CDAI for CD		242.0 (210.0–263.3)	108.5 (91.3–139.3)			<0.001
Mayo for UC				6.0 (4.0–8.0)	2.0 (1.0–2.0)	<0.001

*Data were presented as mean (standard deviation), count or median (inter-quartile range). Difference was determined by one-way analysis of variance (ANOVA), Chi-square test, Wilcoxon rank sum test or Kruskal-Wallis H rank sum test. P < 0.05 was considered significant. IBD, inflammatory bowel disease; HC, healthy controls; A-CD, active Crohn's disease; R-CD, Crohn's disease with remission; A-UC, active ulcerative colitis; R-UC, ulcerative colitis with remission; CRP, C reactive protein; ESR, erythrocyte sedimentation rate; CDAI, Crohn's disease activity index*.

### Correlation of lnc-ITSN1-2 Expression With Disease Activity in IBD Patients

**Figure 3:**
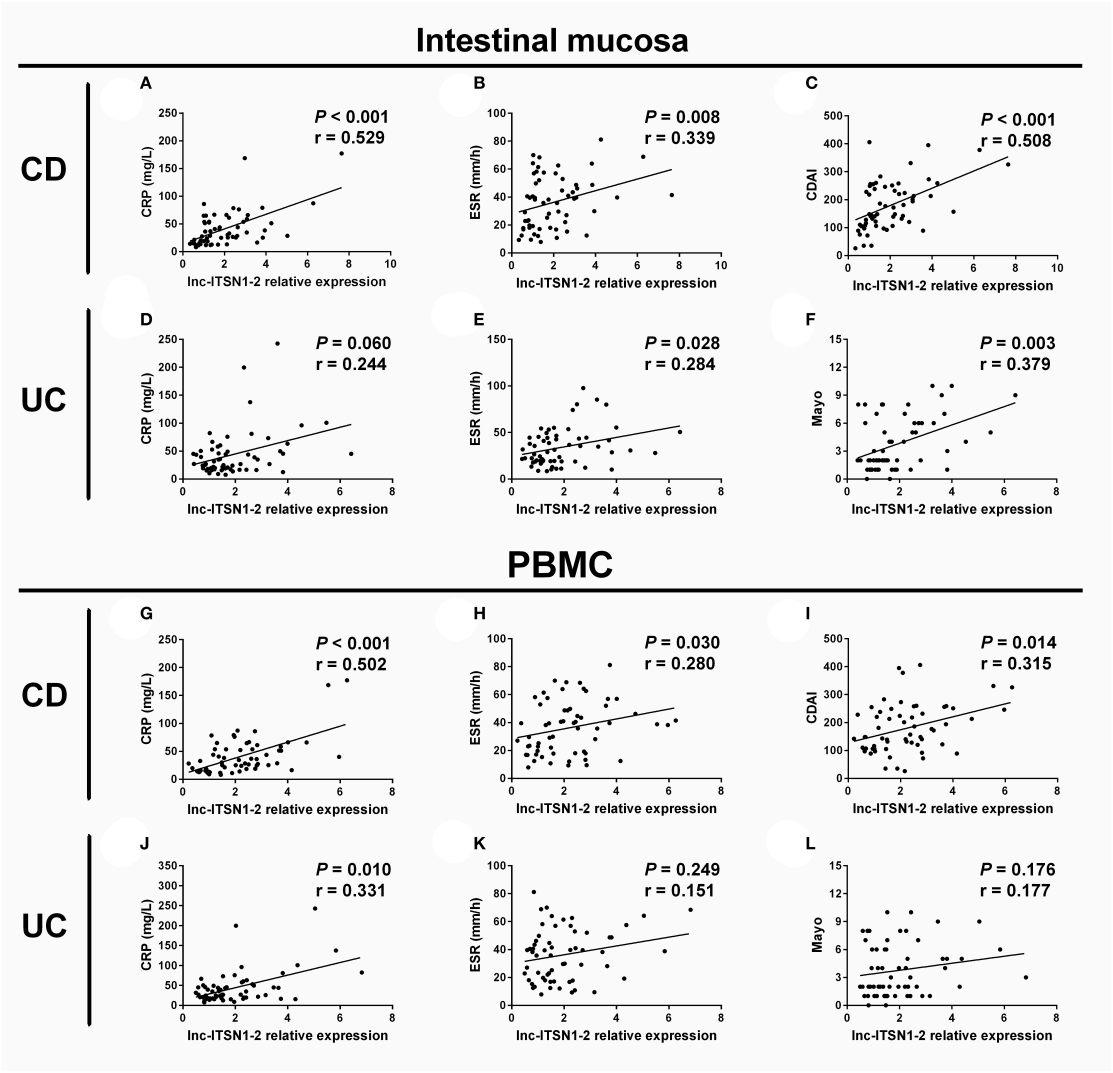
Correlation of lnc-ITSN1-2 with IBD activity. Correlation of intestinal mucosa lnc-ITSN1-2 with CRP **(A)**, ESR **(B)**, and CDAI **(C)** in CD patients; Correlation of intestinal mucosa lnc-ITSN1-2 with CRP **(D)**, ESR **(E)**, and Mayo **(F)** in UC patients; Correlation of PBMC lnc-ITSN1-2 with CRP **(G)**, ESR **(H)**, and CDAI **(I)** in CD patients; Correlation of PBMC lnc-ITSN1-2 with CRP **(J)**, ESR **(K)**, and Mayo **(L)** in UC patients. IBD, inflammatory bowel disease; CRP, C-reactive protein; ESR, erythrocyte sedimentation rate; CDAI, Crohn's disease activity index; CD, Crohn's disease; UC, ulcerative colitis; PBMC, peripheral blood mononuclear cells.

Intestinal mucosa lnc-ITSN1-2 was positively correlated with CRP (Figure 3A), ESR (Figure 3B), and CDAI score (Figure 3C) in CD patients, and was also positively associated with ESR (Figure 3E) and Mayo score (Figure 3F), while it did not correlate with CRP (Figure 3D) in UC patients. As for PBMC lnc-ITSN1-2, it was observed to be positively correlated with CRP (Figure 3G), ESR (Figure 3H), and CDAI score (Figure 3I) in CD patients, and was also positively associated with CRP (Figure 3J) but not the ESR (Figure 3K) and Mayo score (Figure 3L) in UC patients. Taken together, these data suggest that lnc-ITSN1-2 possesses the potential to be a biomarker of IBD disease activity and it might be implicated in the progression of IBD.

### Correlation of lnc-ITSN1-2 Expression With Inflammatory Cytokines Expressions in IBD Patients

**Figure 4:**
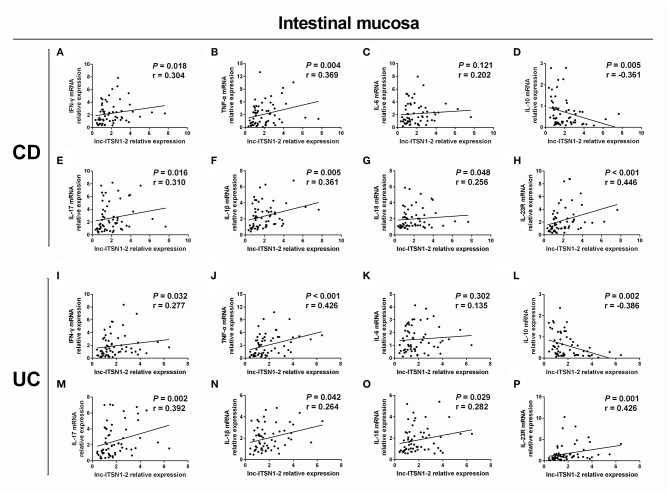
Correlation of intestinal mucosa lnc-ITSN1-2 with inflammatory cytokines and IL-23R. Correlation of intestinal mucosa lnc-ITSN1-2 with INF-γ **(A)**, TNF-α **(B)**, IL-6 **(C)**, IL-10 **(D)**, IL-17 **(E)**, IL-1β **(F)**, IL-18 **(G)**, and IL-23R **(H)** in CD patients; Correlation of intestinal mucosa lnc-ITSN1-2 with INF-γ **(I)**, TNF-α **(J)**, IL-6 **(K)**, IL-10 **(L)**, IL-17 **(M)**, IL-1β **(N)**, IL-18 **(O)**, and IL-23R **(P)** in UC patients. CD, Crohn's disease; UC, ulcerative colitis.

**Figure 5:**
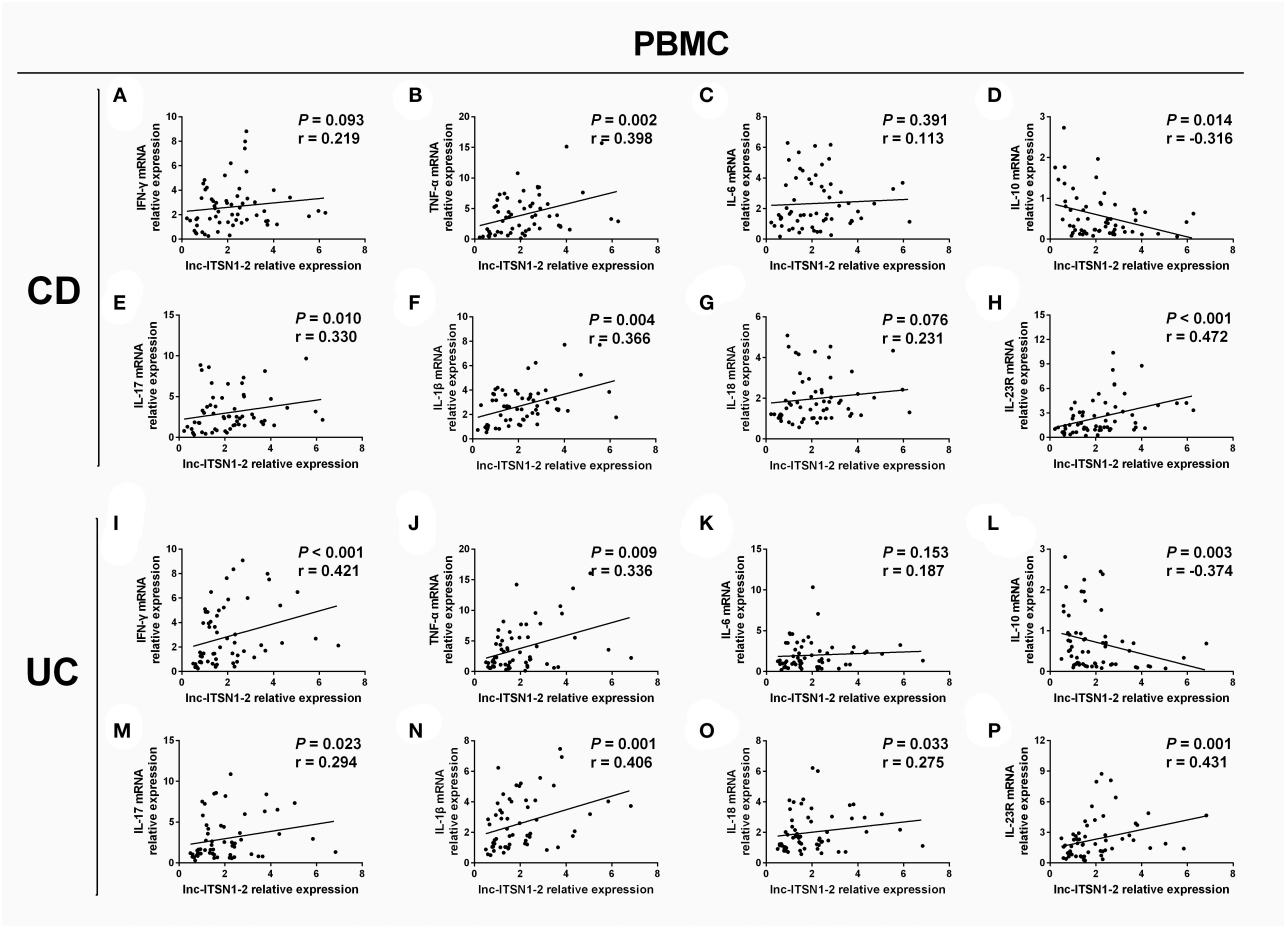
Correlation of PBMC lnc-ITSN1-2 with inflammatory cytokines and IL-23R. Correlation of PBMC lnc-ITSN1-2 with INF-γ **(A)**, TNF-α **(B)**, IL-6 **(C)**, IL-10 **(D)**, IL-17 **(E)**, IL-1β **(F)**, IL-18 **(G)**, and IL-23R **(H)** in CD patients; Correlation of PBMC lnc-ITSN1-2 with INF-γ **(I)**, TNF-α **(J)**, IL-6 **(K)**, IL-10 **(L)**, IL-17 **(M)**, IL-1β **(N)**, IL-18 **(O)**, and IL-23R **(P)** in UC patients. PBMC, peripheral blood mononuclear cells; CD, Crohn's disease; UC, ulcerative colitis.

Intestinal mucosa lnc-ITSN1-2 was positively associated with IFN-γ (Figure 4A), TNF-α (Figure 4B), IL-17 (Figure 4E), IL-1β (Figure 4F), and IL-18 (Figure 4G) and was negatively correlated with IL-10 (Figure 4D), while it did not associate with IL-6 (Figure 4C) in CD patients. Furthermore, it was also positively correlated with IFN-γ (Figure 4I), TNF-α (Figure 4J), IL-17 (Figure 4M), IL-1β (Figure 4N), and IL-18 (Figure 4O), and was negatively correlated with IL-10 (Figure 4L), while it did not associate with IL-6 (Figure 4K) in UC patients. As for PBMC lnc-ITSN1-2, it was positively correlated with TNF-α (Figure 5B), IL-17 (Figure 5E), and IL-1β (Figure 5F), and was negatively correlated with IL-10 (Figure 5D), while it did not correlate with IFN-γ (Figure 5A), IL-6 (Figure 5C), or IL-18 (Figure 5G) in CD patients. Additinoally, it was positively correlated with IFN-γ (Figure 5I), TNF-α (Figure 5J), IL-17 (Figure 5M), IL-1β (Figure 5N), and IL-18 (Figure 5O), and was negatively correlated with IL-10 (Figure 5L), while it did not associate with IL-6 (Figure 5K) in UC patients. Combining these data, lnc-ITSN1-2 was closely correlated with increased inflammation in IBD.

### Correlation of lnc-ITSN1-2 Expression With IL-23R Expression in IBD Patients

Our bioinformatics analysis of RNA sequencing revealed that IL-23R was a key gene regulated by lnc-ITSN1-2 in IBD (Figure 1E), and IL-23R was one of the critical genes implicated in the etiology of IBD (20), thus, we hypothesized that lnc-ITSN1-2 might be involved in IBD pathogenesis via IL-23R. We determined both intestinal mucosa and PBMC IL-23R in IBD patients and observed that intestinal mucosa lnc-ITSN1-2 was positively correlated to IL-23R in CD (Figure 4H) and UC patients (Figure 4P). Similarly, PBMC lnc-ITSN1-2 was also positively associated with IL-23R in CD (Figure 5H) and UC patients (Figure 5P). Furthermore, we also compared IL-23R expression between IBD patients and HCs, which revealed that intestinal mucosa IL-23R was increased in A-CD, R-CD, and A-UC patients, while similar results were revealed in R-UC patients compared to HCs (Supplementary Figure 3A). PBMC IL-23R was increased in A-CD, R-CD, A-UC, and R-UC patients compared to the HCs (Supplementary Figure 3B). These data indicated that lnc-ITSN1-2 might be involved in IBD pathogenesis via regulating IL-23R.

### Correlation of Intestinal Mucosa and PBMC lnc-ITSN1-2 Expressions

**Figure 6:**
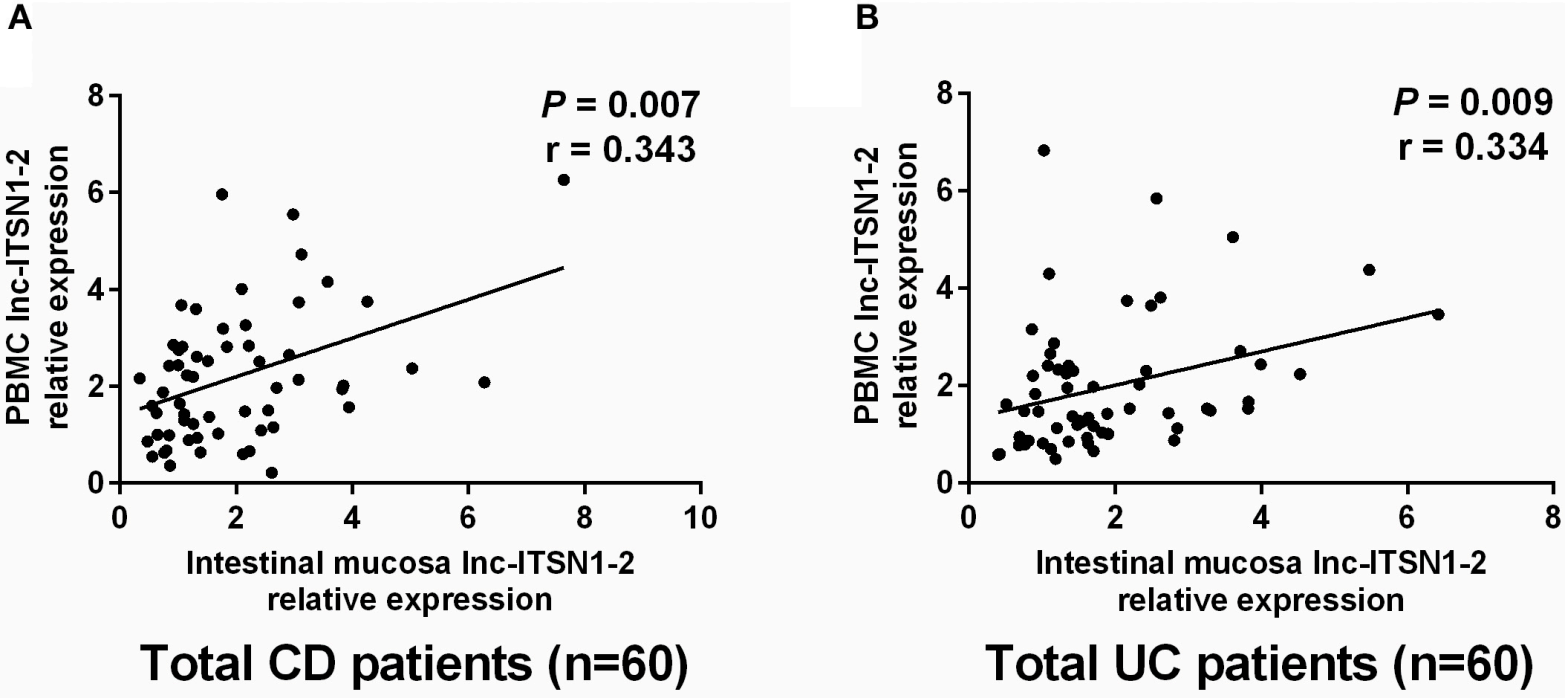
Correlation of intestinal mucosa lnc-ITSN1-2 and PBMC lnc-ITSN1-2. Intestinal mucosa lnc-ITSN1-2 was positively correlated with PBMC lnc-ITSN1-2 in both CD patients **(A)** and UC patients **(B)**. PBMC, peripheral blood mononuclear cells; CD, Crohn's disease; UC, ulcerative colitis.

We then analyzed the correlation of intestinal mucosa and PBMC lnc-ITSN1-2 expressions in CD and UC patients, which disclosed that intestinal mucosa lnc-ITSN1-2 was positively correlated with PBMC lnc-ITSN1-2 in CD (Figure 6A) and UC patients (Figure 6B).

### Change of lnc-ITSN1-2 Expression After IFX Treatment

**Figure 7:**
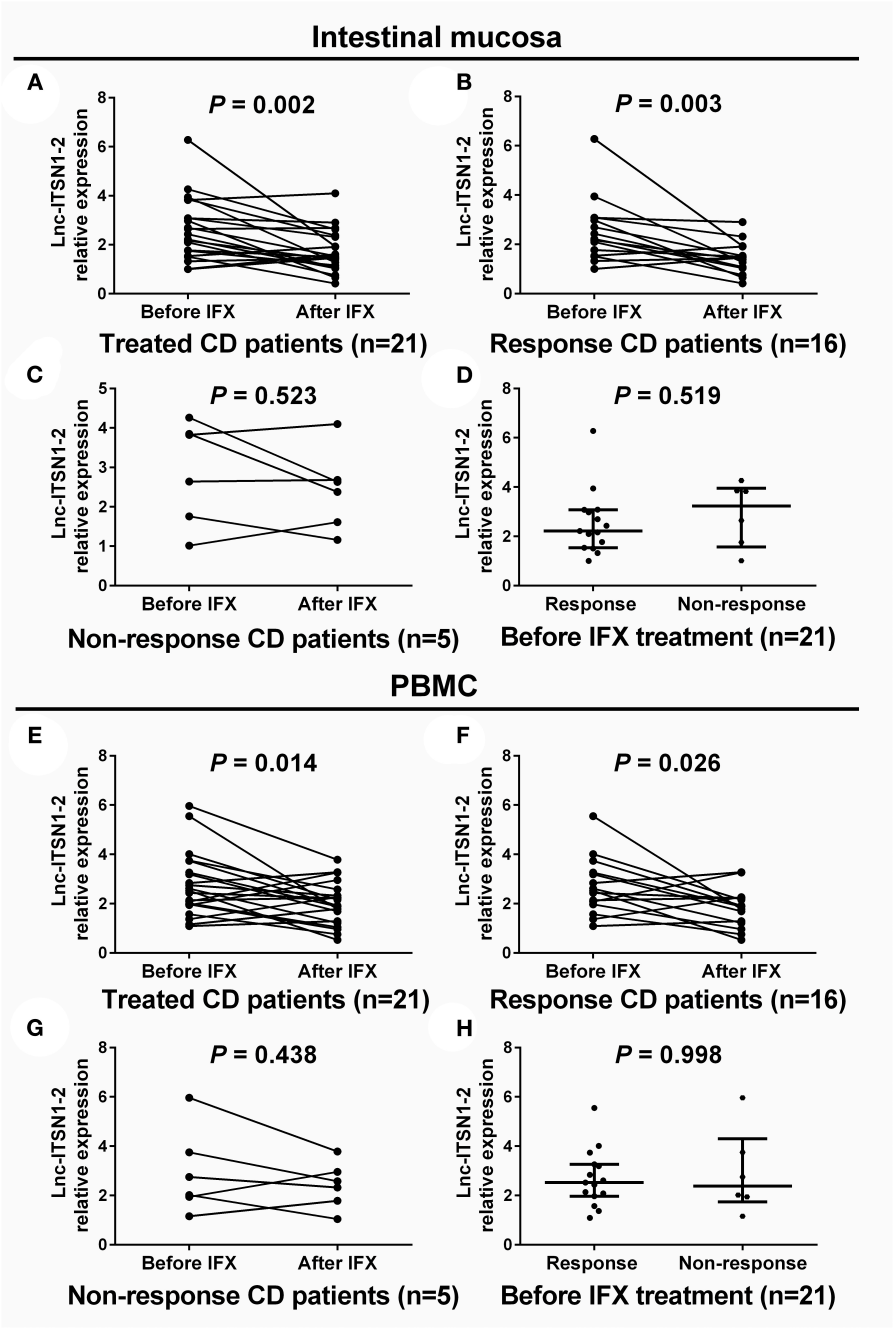
Comparison of lnc-ITSN1-2 before and after IFX treatment. Intestinal mucosa lnc-ITSN1-2 was decreased after IFX treatment in response CD patients but not in non-response CD patients **(A–C)**, and baseline intestinal mucosa lnc-ITSN1-2 was similar between response and non-response CD patients **(D)**. PBMC lnc-ITSN1-2 was decreased after IFX treatment in response CD patients but not in non-response CD patients **(E–G)** as well, and baseline PBMC lnc-ITSN1-2 was of no difference between response and non-response CD patients **(H)**. IFX, infliximab; PBMC, peripheral blood mononuclear cells; CD, Crohn's disease; UC, ulcerative colitis.

IFX treatment was initiated in 21 cases among 30 CD patients. Intestinal mucosa and PBMC lnc-ITSN1-2 expressions were measured at week 0 (before treatment) and week 12 (after treatment). Among the 21 IFX treated A-CD patients, 16 cases achieved a clinical response (response group) while the other five cases failed to achieve a clinical response (non-response group). In the total treated patients, intestinal mucosa lnc-ITSN1-2 was decreased after IFX treatment (Figure 7A). In the response patients, intestinal mucosa lnc-ITSN1-2 was also lower after IFX treatment (Figure 7B), while in the non-response patients, intestinal mucosa lnc-ITSN1-2 remained the same after IFX treatment (Figure 7C). Additionally, baseline intestinal mucosa lnc-ITSN1-2 showed no difference between the response and non-response patients (Figure 7D). PBMC lnc-ITSN1-2 also presented with the same trend (Figures 7E–H). Collectively, these data indicated that IFX treatment could downregulate intestinal mucosa and PBMC lnc-ITSN1-2 in response CD patients, which implied that the change of lnc-ITSN1-2 correlated with CD treatment efficacy.

### Effect of lnc-ITSN1-2 on IBD CD4^+^ T Cell Activation

**Figure 8:**
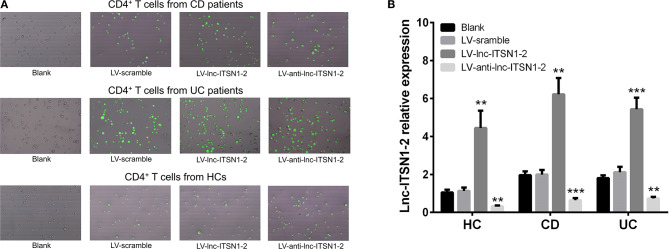
Lnc-ITSN1-2 expression in CD4^+^ T cells after transfection. **(A)** Presented examples of transfection images. **(B)** Comparison of lnc-ITSN1-2 expression in each group. ^**^P < 0.01, ^***^P < 0.001. HC, health control; CD, Crohn's disease; UC, ulcerative colitis.

**Figure 9:**
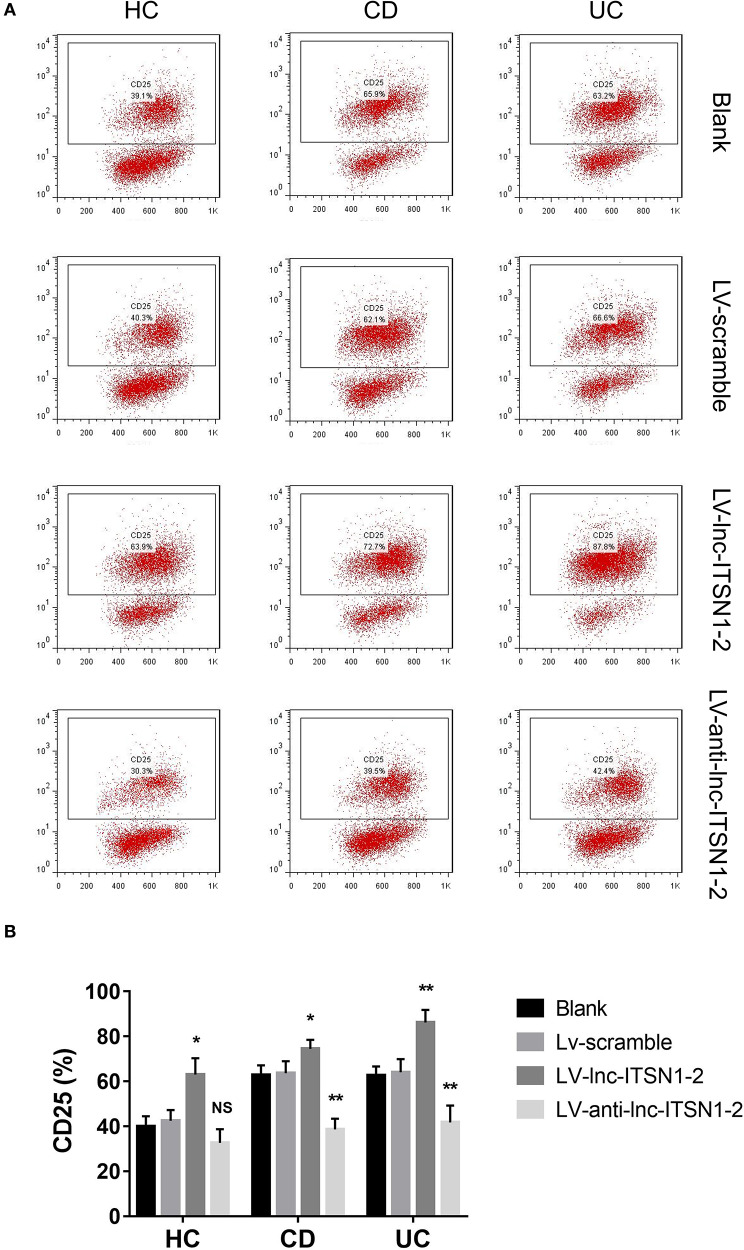
CD25^+^ cell percentage after transfection. **(A)** Presented examples of CD25^+^ cell percentage images. **(B)** Comparison of CD25^+^ cell percentage in each group. NS, *P* > 0.05; ^*^P < 0.05; ^**^P < 0.01. HC, health control; CD, Crohn's disease; UC, ulcerative colitis.

**Figure 10:**
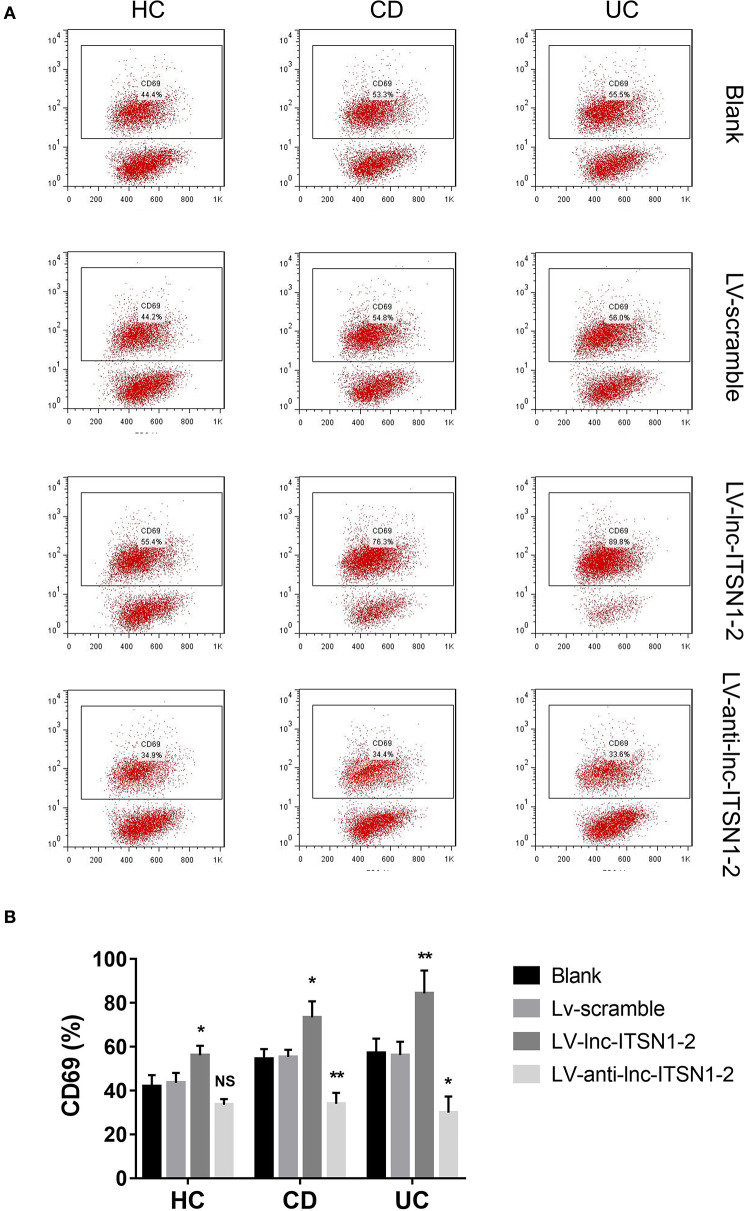
CD69+ cell percentage after transfection. **(A)** Presented examples of CD69^+^ cell percentage images. **(B)** Comparison of CD69+ cell percentage in each group. NS, *P* > 0.05; ^*^P < 0.05; ^**^P < 0.01. HC, health control; CD, Crohn's disease; UC, ulcerative colitis.

Since both intestinal mucosa and PBMC lnc-ITSN1-2 closely correlated with risk, severity, and inflammation response of IBD, and since CD4^+^ T cell activation, proliferation as well as differentiation were key bioprocesses of IBD development and progression, we hypothesized that lnc-ITSN1-2 might regulate IBD CD4^+^ T cell activation, proliferation, and differentiation, and we thus carried out the following *in vitro* experiments. Figure 8A presented the transfection images examples of the Blank group, LV-scramble group, LV-lnc-ITSN1-2 group, and the LV-anti-lnc-ITSN1-2 group in IBD and HC CD4^+^ T cells. lnc-ITSN1-2 was discovered to be upregulated in the LV-lnc-ITSN1-2 group while downregulated in the LV-anti-lnc-ITSN1-2 group compared to the LV-scramble group (Figure 8B). For detection of IBD CD4^+^ T cell activation, we found that CD25^+^ cell percentage (Figures 9A,B) and CD69^+^ cell percentage (Figures 10A,B) were both elevated in the LV-lnc-ITSN1-2 group while reduced in the LV-anti-lnc-ITSN1-2 group compared to the LV-scramble group. While for HC CD4^+^ T cell activation, we found that CD25^+^ cell percentage (Figures 9A,B) and CD69^+^ cell percentage (Figures 10A,B) were higher in the LV-lnc-ITSN1-2 but similar in the LV-anti-lnc-ITSN1-2 group compared to the LV-scramble group. These data suggested that lnc-ITSN1-2 promoted IBD CD4^+^ T cell activation.

### Effect of lnc-ITSN1-2 on IBD CD4^+^ T Cell Proliferation

**Figure 11:**
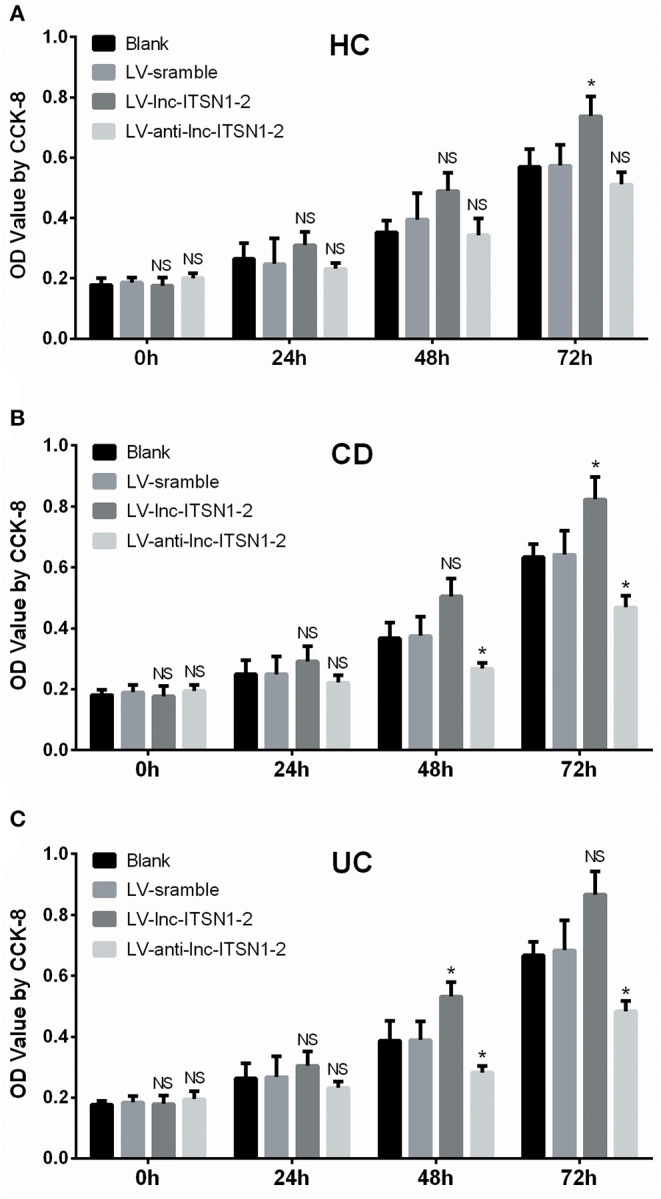
CD4^+^ T cell proliferation after transfection. Comparison of CD4^+^ T cell proliferation (assessed by OD value using CCK-8) among groups at 0, 24, 48, and 72 h. **(A)** HC CD4^+^ T cells; **(B)** CD CD4^+^ T cells; **(C)**. UC CD4^+^ T cells. NS, *P* > 0.05; ^*^P < 0.05. HC, health control; CD, Crohn's disease; UC, ulcerative colitis.

HC CD4^+^ T cell proliferation was only increased at 72 h in the LV-lnc-ITSN1-2 group compared to the LV-scramble group, but was of no difference in the LV-anti-lnc-ITSN1-2 group compared to the LV-scramble group (Figure 11A). Notably, proliferation of the CD4^+^ T cell, derived from CD patients, was observed to be increased at 72 h in the LV-lnc-ITSN1-2 group, while being decreased at 48 h and 72 h in the LV-anti-lnc-ITSN1-2 group compared to the LV-scramble group (Figure 11B). Proliferation of the CD4^+^ T cell, derived from UC patients, was observed to be increased at 48 h in the LV-lnc-ITSN1-2 group, while being decreased at 48 and 72 h in the LV-anti-lnc-ITSN1-2 group compared to the LV-scramble group (Figure 11C). These data indicated that lnc-ITSN1-2 enhanced IBD CD4^+^ T cell proliferation.

### Effect of lnc-ITSN1-2 on IBD CD4^+^ T Cell Differentiation

**Figure 12:**
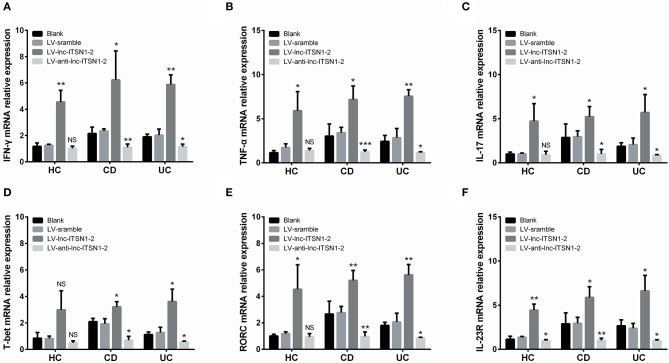
CD4^+^ T cell differentiation and IL-23R after transfection. Comparison of IFN-γ **(A)**, TNF-α **(B)**, IL-17 **(C)**, T-bet **(D)**, RORC **(E)**, and IL-23R **(F)** among groups. NS, *P* > 0.05; ^*^P < 0.05; ^**^P < 0.01. HC, health control; CD, Crohn's disease; UC, ulcerative colitis.

IFN-γ, TNF-α, IL-17, T-bet, and RORC expressions in IBD CD4^+^ T cells were all increased in the LV-lnc-ITSN1-2 group while decreased in the LV-anti-lnc-ITSN1-2 group compared to the LV-scramble group (Figures 12A–E). However, only IFN-γ, TNF-α, IL-17, and RORC expressions in HC CD4^+^ T cells were increased in the LV-lnc-ITSN1-2 group compared to the LV-scramble group, while no difference of IFN-γ, TNF-α, IL-17, T-bet, or RORC expression was observed in the LV-anti-lnc-ITSN1-2 group and the LV-scramble group (Figures 12A–E). These data implied that lnc-ITSN1-2 improved IBD Th1/Th17 cell differentiation.

### Lnc-ITSN1-2 Regulated IL-23R in IBD CD4^+^ T Cells

Our bioinformatics analysis of RNA sequencing revealed that IL-23R was a key gene regulated by lnc-ITSN1-2 in IBD (Figure 1E), and IL-23R was positively correlated with lnc-ITSN1-2 in a large-sample validation by RT-qPCR (Figures 4F,L, 5F,L). In addition, IL-23R was one of the reported critical genes implicated in the etiology of IBD (20). We further analyzed IL-23R expression after lnc-ITSN1-2 lentivirus transfection in IBD CD4^+^ T cells, and found that IL-23R was upregulated in the LV-lnc-ITSN1-2 group while decreased in the LV-anti-lnc-ITSN1-2 group compared to the LV-scramble group (Figure 12F).

### Lnc-ITSN1-2 Functioned in IBD CD4^+^ T Cells via Regulating IL-23R

**Figure 13:**
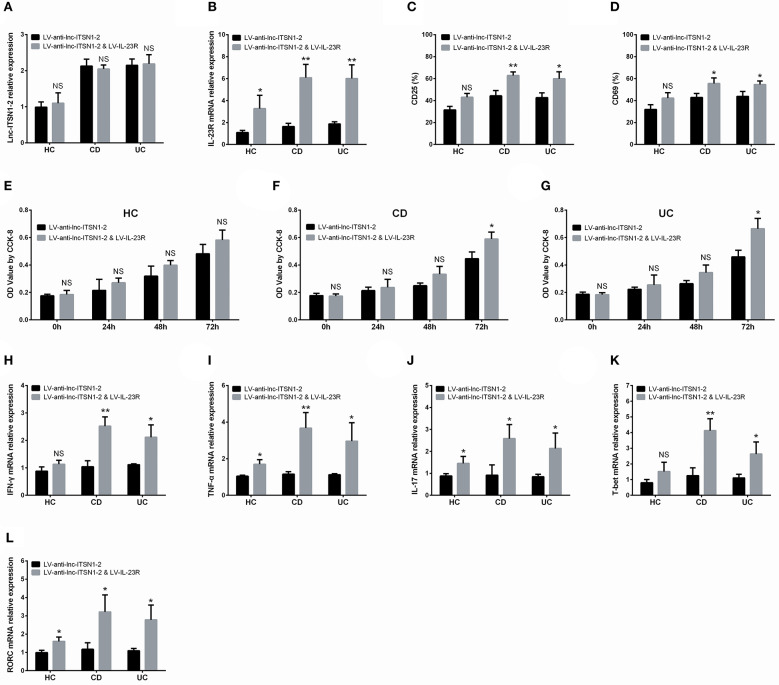
Rescue experiment: IL-23R overexpression in lnc-ITSN1-2 knockdown treated CD4^+^ T cells. Comparison of lnc-ITSN1-2 expression **(A)**, IL-23R expression **(B)**, CD25^+^ cell percentage **(C)**, CD69^+^ cell percentage **(D)**, CD4^+^ T cell proliferation **(E–G)**, IFN-γ expression **(H)**, TNF-α expression **(I)**, IL-17 expression **(J)**, T-bet expression **(K)**, RORC expression **(L)** between LV-anti-lnc-ITSN1-2 & LV-IL-23R group and LV-anti-lnc-ITSN1-2 group. NS, *P* > 0.05; ^*^P < 0.05; ^**^P < 0.01. HC, health control; CD, Crohn's disease; UC, ulcerative colitis.

Based on the data presented above, we then hypothesized that lnc-ITSN1-2 might function in IBD CD4^+^ T cells via regulating IL-23R, thus subsequent rescue experiments were performed. We observed that in IBD CD4^+^ T cells, lnc-ITSN1-2 expression was similar (Figure 13A), while IL-23R expression (Figure 13B) was increased in the LV-anti-lnc-ITSN1-2 & LV-IL-23R group compared to the LV-anti-lnc-ITSN1-2 group. Notably, CD25^+^ cell percentage, CD69^+^ cell percentage, cell proliferation, IFN-γ, TNF-α, IL-17, T-bet, and RORC expressions were increased in the LV-anti-lnc-ITSN1-2 & LV-IL-23R group compared to the LV-anti-lnc-ITSN1-2 group (Figures 13C–L). However, in HC CD4^+^ T cells, less difference of the abovementioned indexes was observed in the LV-anti-lnc-ITSN1-2 & LV-IL-23R group compared to the LV-anti-lnc-ITSN1-2 group (Figures 13A–E,H–L). Furthermore, we observed that lnc-ITSN1-2 knockdown decreased the pJAK2, pSTAT3, IKKA, and NF-κB expressions in both CD and UC CD4^+^ T cells, while IL-23R overexpression improved the pJAK2, pSTAT3, IKKA, and NF-κB expressions in lnc-ITSN1-2 knockdown treated CD and UC CD4^+^ T cells (Supplementary Figures 4A,B), which indicated that the JAK2/STAT3 pathway and NF-κB pathway were regulated by lnc-ITSN1-2 through IL-23R in IBD CD4^+^ T cells.

### Lnc-ITSN1-2 Targeted miR-125a and Served as Competing Endogenous RNA (ceRNA) for IL-23R, Contributing to Its Regulation of IBD CD4^+^ T Cells

Based on target prediction using miRanda Database (www.microrna.org), and the anti-inflammation role of miR-125a as well as the pro-inflammation role of IL-23R, we further explored whether lnc-ITSN1-2 regulated the miR-125a/IL-23R pathway to regulate IBD CD4^+^ T cells. First, we observed that lnc-ITSN1-2 negatively regulated miR-125a in IBD and HC CD4^+^ T cells (Supplementary Figure 5A), then rescue experiments showed that miR-125a knockdown increased CD25^+^ cell percentage, CD69^+^ cell percentage, cell proliferation, and IFN-γ, TNF-α, IL-17, and T-bet as well as RORC expressions in lnc-ITSN1-2 knockdown treated IBD CD4^+^ T cells (Supplementary Figures 5B–M). However, the attenuating effect of miR-125a knockdown was not obvious in lnc-ITSN1-2 knockdown treated HC CD4^+^ T cells (Supplementary Figures 5B–M). Second, we observed that miR-125a negatively regulated IL-23R in IBD and HC CD4^+^ T cells (Supplementary Figure 6A). Then rescue experiments showed that IL-23R knockdown decreased CD25^+^ cell percentage, CD69^+^ cell percentage, cell proliferation, and IFN-γ, TNF-α, IL-17, and T-bet as well as RORC expressions in miR-125a knockdown treated IBD CD4^+^ T cells (Supplementary Figures 6B–M). However, the compensation effect of IL-23R knockdown was not obvious in miR-125a knockdown treated HC CD4^+^ T cells (Supplementary Figures 6B–M). Third, Luciferase Reporter assays were performed, and we found that lnc-ITSN1-2 directly bound to miR-125a (Supplementary Figures 7A,B), and miR-125a directly bound to IL-23R (Supplementary Figures 7C,D). Combining this together, lnc-ITSN1-2 promoted IBD CD4^+^ T cell activation and proliferation, and stimulated Th1/Th17 cell differentiation through sponging miR-125a to positively regulate IL-23R (lnc-ITSN1-2 serves as ceRNA).

## Discussion

In this present study, we observed that: (1) 309 upregulated lncRNAs and 310 downregulated lncRNAs were identified in IBD patients by RNA sequencing, which were enriched in immune and inflammation related pathways; (2) Both intestinal mucosa and PBMC lnc-ITSN1-2 expressions are increased in IBD patients compared to HCs, by RT-qPCR validation, which presented with a good predictive value for IBD risk—especially for active disease condition—and they positively correlated with disease activity and inflammation indexes in IBD patients; (3) Most importantly, lnc-ITSN1-2 promoted IBD CD4^+^ T cell activation and proliferation, and stimulated Th1/Th17 cell differentiation by regulating IL-23R; (4) Further mechanism investigations observed that lnc-ITSN1-2 directly sponged miR-125a to positively regulate IL-23R (lnc-ITSN1-2 serves as ceRNA), via this regulation, and lnc-ITSN1-2 regulated CD4^+^ T cell functions in IBD.

LncRNAs, along with the fast progress in second-generation of sequencing, have attracted a lot of attention in recent years. Although lncRNAs lack protein-coding ability, they have been discovered to participate in various molecule regulations and bioprocesses through epigenetic regulation, gene imprinting, transcriptional activation, mRNA modification, nuclear transportation, and protein viability activation (7, 12). lncRNAs are also observed to be involved in the etiology of many diseases including cancers, vascular disease, inflammatory diseases, and so on (7, 8, 21). A previous study discovered 683 upregulated and 1416 downregulated lncRNAs in PBMC of RA patients compared to HCs, which are enriched in regulating viral myocarditis, rheumatoid arthritis development, and glycosphingolipid biosynthesis (22). Another study reports that 3657 lncRNAs are upregulated and 4014 lncRNAs are downregulated in PBMC of SLE patients compared to controls (23). These indicate that lncRNAs are implicated in the etiology of inflammatory and immune diseases. As for IBD lncRNA expression profiles, few studies have been reported, and only one recent study reports that 438 and 745 lncRNAs are dysregulated in Denmark CD and UC patients compared with controls, respectively, and these dysregulated lncRNA are enriched in regulating immune response, pro-inflammatory cytokine activity, and natural killer cell activation (24). However, this study does not filter genes with less occurrence in samples or adjust *P*-value, which might cause a bias in the result, and the patients recruited in this study are of a white population, thus the lncRNA expression profiles in Chinses IBD patients remain unknown (24). In this present study, we detected lncRNA expression profiles in Chinses IBD patients as well as HCs, and 309 upregulated lncRNAs and 310 downregulated lncRNAs were identified in intestinal mucosa samples from six IBD patients compared to six HCs by RNA sequencing, which were enriched in regulating immune and inflammation related pathways These implied that dysregulated lncRNA expression profiles are implicated in the pathogenesis of IBD.

Benefiting from the high conservation and stability, a number of lncRNAs have recently been discovered to be novel biomarkers for disease risk and severity monitoring. For instance, a study identified five lncRNAs (RNA143598, RNA143596, HIX0032090, IGHCgamma1, and XLOC_002730) that are upregulated in serum of RA patients compared to HCs, which are also positively associated with disease duration, ESR, RF positive, or ACPA positive (25). Another study discovered that lncRNA TUG1 is greatly lower in ankylosing spondylitis patients compared to controls and correlates with decreased disease activity, a short course of treatment, and a reduced rehospitalization rate (26). These suggest the potential of lncRNA as a novel biomarker for inflammatory diseases, while in studies investing lncRNAs as a biomarker for IBD, very limited reports have been disclosed. A previous study exhibits that colonic mucosa lncRNA ANRIL is highly expressed in UC patients compared to HCs, while its diagnostic value for UC and correlation of disease severity is not evaluated (13). Another study discovered that lncRNA DQ786243 is upregulated in active CD patients compared with CD patients in remission and HCs, while its diagnostic value for CD and the association of disease activity was not analyzed either (11). Considering that lnc-ITSN1-2 was one of the most dysregulated DELs in IBD patients compared to HCs by RNA sequencing in our study, and because it is also reported to be implicated in development and progression of RA (15), a potential biomarker for coronary artery disease risk (although lacks significance, but presents with a trend) (16), we hypothesized that lnc-ITSN1-2 might be involved in IBD pathogenesis. Thus, we further validated lnc-ITSN1-2 expression in both intestinal mucosa and PBMC samples from 120 IBD (including 30 A-CD, 30 R-CD, 30 A-UC, and 30 R-UC) patients and 30 HCs using RT-qPCR. In this present study, we observed that both intestinal mucosa and PBMC lnc-ITSN1-2 expressions were increased in IBD patients compared to HCs, which presented with good predictive value for IBD risk by ROC curve analysis, and they positively correlated with disease activity and inflammation indexes in IBD patients. The possible explanations were as follows: (1) lnc-ITSN1-2 positively regulated IL-23R (validated in our following *in vitro* experiments) which was a key regulator of immune response and inflammation, thus lnc-ITSN1-2 was upregulated and positively correlated with disease activity and inflammation indexes in IBD patients. (2) lnc-ITSN1-2 promoted IBD CD4^+^ T cell activation, proliferation, and stimulated Th1/Th17 cell differentiation (validate in our followed *in vitro* experiments), resulting in increased inflammation, thus lnc-ITSN1-2 was upregulated and positively correlated with disease activity and inflammation indexes in IBD patients. Furthermore, there is doubt as to which factors might contribute to lnc-ITSN1-2 induction. Two previous studies observe that rheumatoid arthritis patients present with increased lnc-ITSN1-2 expression in blood and synovial tissue, along with a positive correlation with systemic inflammation (15, 27), which is partially in line with our results that lnc-ITSN1-2 was raised in IBD patients and correlated with higher systemic or lesion site inflammation. Therefore, we speculated that increased inflammation and hyper-proliferation of intestinal mucosa, and the activated blood CD4^+^ T cells might contribute to the induction of lnc-ITSN1-2. However, in this study, we only performed qPCR to detect gene expressions, which was due to the fact that: (1) lnc-ITSN1-2 was observed to serve as a ceRNA for IL-23R via binding miR-125a in our subsequent *in vitro* experiments, which meant lnc-ITSN1-2 functioned in IBD based on RNA dimensions; however, (2) an insufficient budget limited our application of ELISA and antibodies purchasing for this experiment.

Interestingly, we also observed that both intestinal mucosa and PBMC lnc-ITSN1-2 were obviously decreased in response CD patients but remained similar in non-response CD patients after a 12-week IFX treatment. The possible explanations are as follows: (1) lnc-ITSN1-2 reflected both intestinal mucosa inflammation and systemic inflammation, and its decrease meant attenuation of inflammation. Therefore, patients with its decrement would be more likely to achieve a clinical response. (2) Based on our subsequent *in vitro* experiments, we speculated that there might be a negative feedback between lnc-ITSN1-2 and TNF-α in HCs but is missed in IBD patients. Therefore, anti-TNF treatment (IFX) would decrease TNF-α then reduce lnc-ITSN1-2 expression in CD patients; Furthermore, the decrease of TNF-α would reflect the treatment response of IFX to some extent.

Accumulating evidence has uncovered that CD4^+^ T cells are critical for the development and progression of chronic intestinal inflammation, and CD4^+^ T cell-related cytokines (such as TNF-α, IFN-γ, IL-1β, and IL-17) are upregulated in the inflamed mucosa of patients with IBD (28, 29). However, the precise mechanisms of how CD4+ T cells are regulated in IBD are still unclear. A previous study observes that 2258 lncRNAs are dysregulated in CD4^+^ T cells derived from neurosyphilis patients compared to controls, and an enrichment analysis revealed these lncRNAs T cell receptors, MAPK, and TGF-β signaling pathways (30). Another study presents that lncRNA IFNG-AS1 promotes CD4^+^ T cell activation and Th1/Treg cell proliferation via regulating HLA-DRB1 in myasthenia gravis (31). Additionally, a recent study also speculates that upregulation of lncRNA TMEVPG1 enhances the Th1 cell response in patients with Sjögren syndrome (32). As to the effect of lnc-ITSN1-2 on regulation of CD4^+^ T cells, no report has been disclosed. In regard to the fact that both intestinal mucosa and PBMC lnc-ITSN1-2 are found to be closely correlated with risk, severity, and inflammation response of IBD in our study, and CD4^+^ T cell activation, proliferation and differentiation are key bioprocesses of IBD development and progression, we hypothesized that lnc-ITSN1-2 might regulate IBD CD4^+^ T cell functions. Thus, we performed *in vitro* experiments, which showed that lnc-ITSN1-2 promoted IBD CD4^+^ T cell activation and proliferation, and stimulated Th1/Th17 cell differentiation through regulating IL-23R. Furthermore, referring to the target prediction using miRanda Database (www.microrna.org), and the ant-inflammation role of miR-125a, as well as the pro-inflammation role of IL-23R, we further explored whether lnc-ITSN1-2 regulated IL-23R via sponging miR-125a to regulate IBD CD4^+^ T cells functions, and these mechanism investigations observed that lnc-ITSN1-2 served as a ceRNA for IL-23R via sponging miR-125a, then regulated CD4+ T cell functions in IBD. However, IL-23R should be activated by IL-23 stimulation, while IL-23 has been reported to be greatly increased in IBD, therefore, we did not perform IL-23 stimulation in the *in vitro* experiments. Furthermore, it was our concern that IL-23 stimulation would affect the induction of lnc-ITSN1-2 and interfere with the assessment of lnc-ITSN1-2 functions in IBD. As to the IL-23 level in IBD patients, we did not determine it, because 100 of reports have reported that IL-23 is greatly increased in IBD patients, and anti-IL-23- antibody [ustekinumab (anti-IL-12&IL-23 antibody)] has already been considered a novel biological treatment in IBD (33, 34). Furthermore, IL-23R is observed to regulate the JAK/STAT signaling pathway derived from the KEGG database (https://www.kegg.jp/) and from several reported studies (35–37). The JAK/STAT pathway is also a well-known pathway which is involved in pathogenesis of IBD, thus, we further speculated that lnc-ITSN1-2 might promote IBD CD4^+^ T cell functions via regulating the IL-23R and downstream JAK/STAT pathway. Our subsequent study observed that the JAK2/STAT3 pathway and NF-κB pathway were indeed regulated by Lnc-ITSN1-2 through IL-23R in IBD CD4+ T cells. These data provided novel evidence for the mechanism of lncRNA as a regulator of CD4^+^ T cell functions, which uncovered a new landscape of IBD pathogenesis.

In conclusion, lncRNA expression profiles play critical roles in the pathogenesis of IBD, and lnc-ITSN1-2 correlates with increased disease risk, activity, and inflammatory cytokines of IBD, and also promotes IBD CD4^+^ T cell activation, proliferation, and Th1/Th17 cell differentiation by serving as a ceRNA for IL-23R via sponging miR-125a.

## Data Availability Statement

The original contributions presented in the study are included in the article/Supplementary Material, further inquiries can be directed to the corresponding author/s.

## Ethics Statement

This study was approved by the Ethics Committee of Zhongnan Hospital of Wuhan University and conducted in accordance with the Declaration of Helsinki, all participants provided written informed consent.

## Author Contributions

JN and QZ conceived this study and collected, investigated, and analyzed the data. All authors approved the final version of the manuscript and agree to be accountable for all aspects of the work in ensuring that questions related to the accuracy or integrity of any part of the work are appropriately investigated and resolved.

## Conflict of Interest

The authors declare that the research was conducted in the absence of any commercial or financial relationships that could be construed as a potential conflict of interest.
