# Supplementary material for: Lnc-ITSN1-2, Derived From RNA Sequencing, Correlates With Increased Disease Risk, Activity and Promotes CD4+ T Cell Activation, Proliferation and Th1/Th17 Cell Differentiation by Serving as a ceRNA for IL-23R via Sponging miR-125a in Inflammatory Bowel Disease
Source: Front Immunol. 2020 May 28;11:852. doi: 10.3389/fimmu.2020.00852 (PMC7271921; doi:10.3389/fimmu.2020.00852)
Supplement: Supplementary file 1 [file Presentation_1.pdf]

Supplementary Material

Supplementary Table 1. Antibodies list.

Antibody	Company	Dilution
Primary Antibody		
Rabbit monoclonal to JAK2	Abcam (UK)	1:5000
Rabbit monoclonal to p-JAK2	Abcam (UK)	1:5000
Rabbit monoclonal to STAT3	Abcam (UK)	1:1000
Rabbit monoclonal to p-STAT3	Abcam (UK)	1:10000
Rabbit monoclonal to IKK alpha (IKKA)	Abcam (UK)	1:20000
Rabbit monoclonal to NFKB	Abcam (UK)	1:5000
Rabbit monoclonal to GAPDH	Abcam (UK)	1:10000
Secondary Antibody		
Goat Anti-Rabbit IgG H&L (HRP)	Abcam (UK)	1:10000

Supplementary Table 2. Primers list.

Gene	Species	Sequence (5'–3')	Sequence (5'-3')
lnc-ITSN1-2	Human	GCTTCACTCGCTTGCTTACA	GGTTCTGTCTTGCCTTCTGTT
IL-23R	Human	TTAGAGACAGAAGAAGAGCAACAG	GCAGAAGGTATCACTATATCATCCA
IFN-γ	Human	TCGGTAACTGACTTGAATGTCCA	TCGCTTCCCTGTTTTAGCTGC
TNF-α	Human	CCTCTCTCTAATCAGCCCTCTG	GAGGACCTGGGAGTAGATGAG
IL-6	Human	CTTCGGTCCAGTTGCCTTCTC	AGGTGAGTGGCTGTCTGTGT
IL-10	Human	AGGGCACCCAGTCTGAGAACA	CGGCCTTGCTCTTGTTTTCAC
IL-17	Human	TCCCACGAAATCCAGGATGC	GGATGTTCAGGTTGACCATCAC
IL-1β	Human	ATGATGGCTTATTACAGTGGCAATG	AGTGGTGGTCGGAGATTCGTA
IL-18	Human	CAAGTTCTCTTCATTGACCAAGGAA	TCACAGAGATAGTTACAGCCATACC
T-bet	Human	GGTTGCGGAGACATGCTGA	GTAGGCGTAGGCTCCAAGG
RORC	Human	GTGGGGACAAGTCGTCTGG	AGTGCTGGCATCGGTTTCG
GAPDH	Human	GGAGCGAGATCCCTCCAAAAT	GGCTGTTGTCATACTTCTCATGG

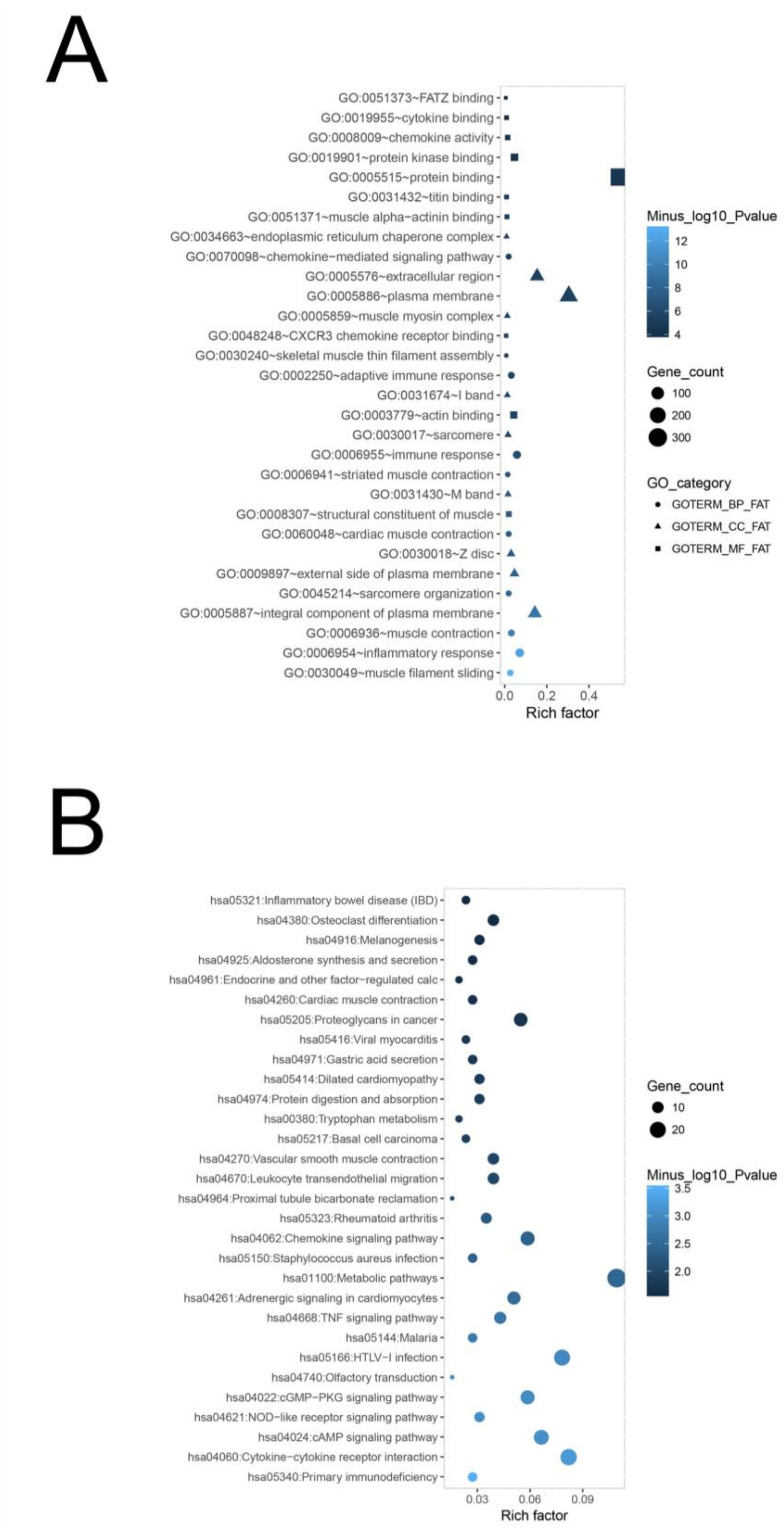

Supplementary figure 1. Enrichment analysis. A. GO enrichment analysis for DELs; B. KEGG enrichment analysis for DELs. DELs, differentially expressed lncRNAs; GO, Gene Ontology; KEGG, Kyoko Encyclopedia of Genes and Genomes.

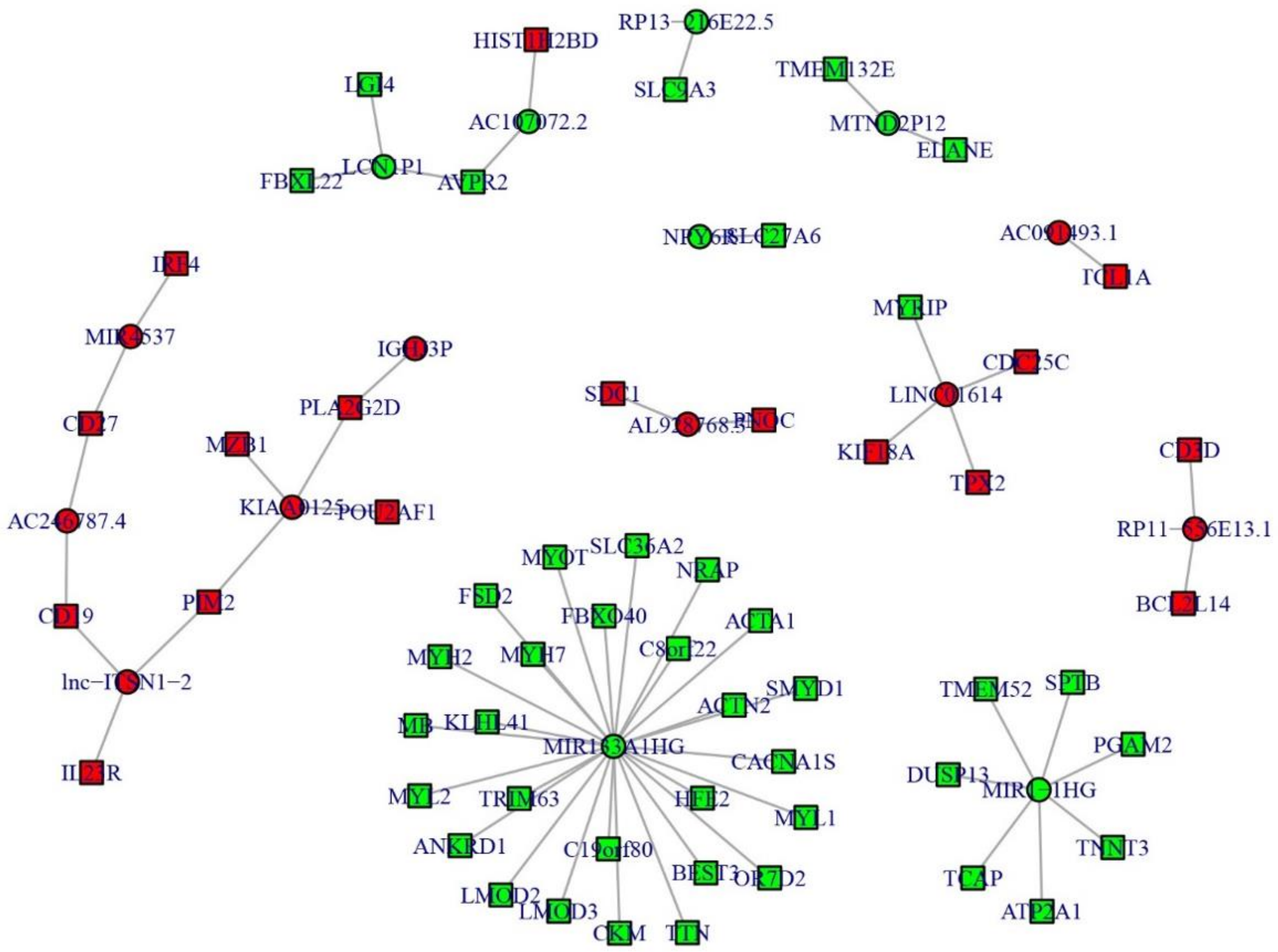

Supplementary figure 2. Regulatory network of top DELs. DELs, differentially expressed lncRNAs.

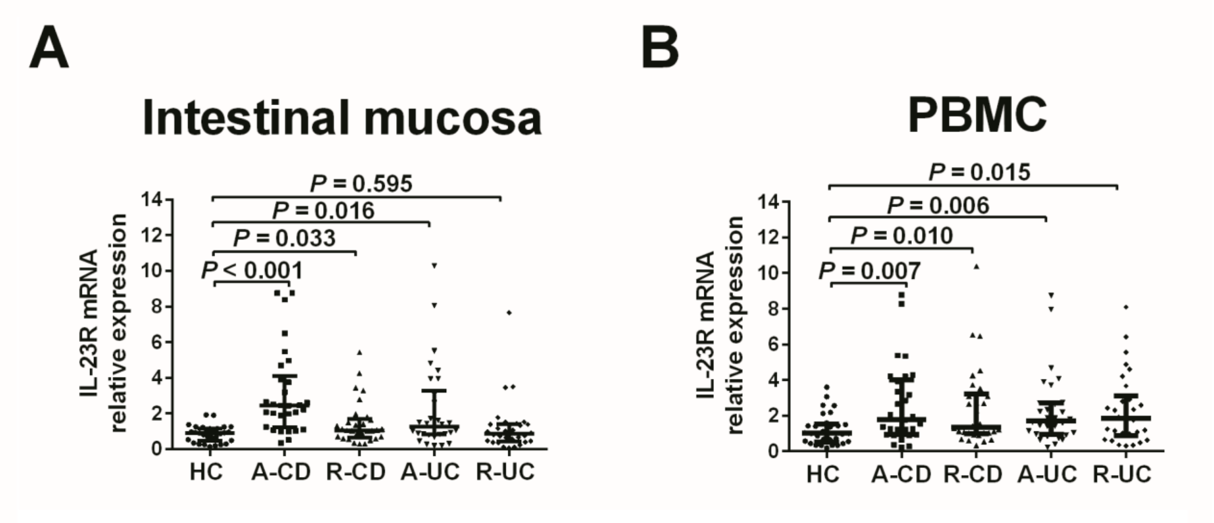

Supplementary figure 3. IL-23R in IBD patients and HCs. A. Comparison of intestinal mucosa IL-23R among HC, A-CD, R-CD, A-UC and R-UC; B. Comparison of PBMC IL-23R among HC, A-CD, R-CD, A-UC and R-UC. IBD, inflammatory bowel disease; HC, health control; A-CD, active Crohn's disease; R-CD, Crohn's disease with remission; A-UC, active ulcerative colitis; R-UC, ulcerative colitis with remission; PBMC, peripheral blood mononuclear cells.

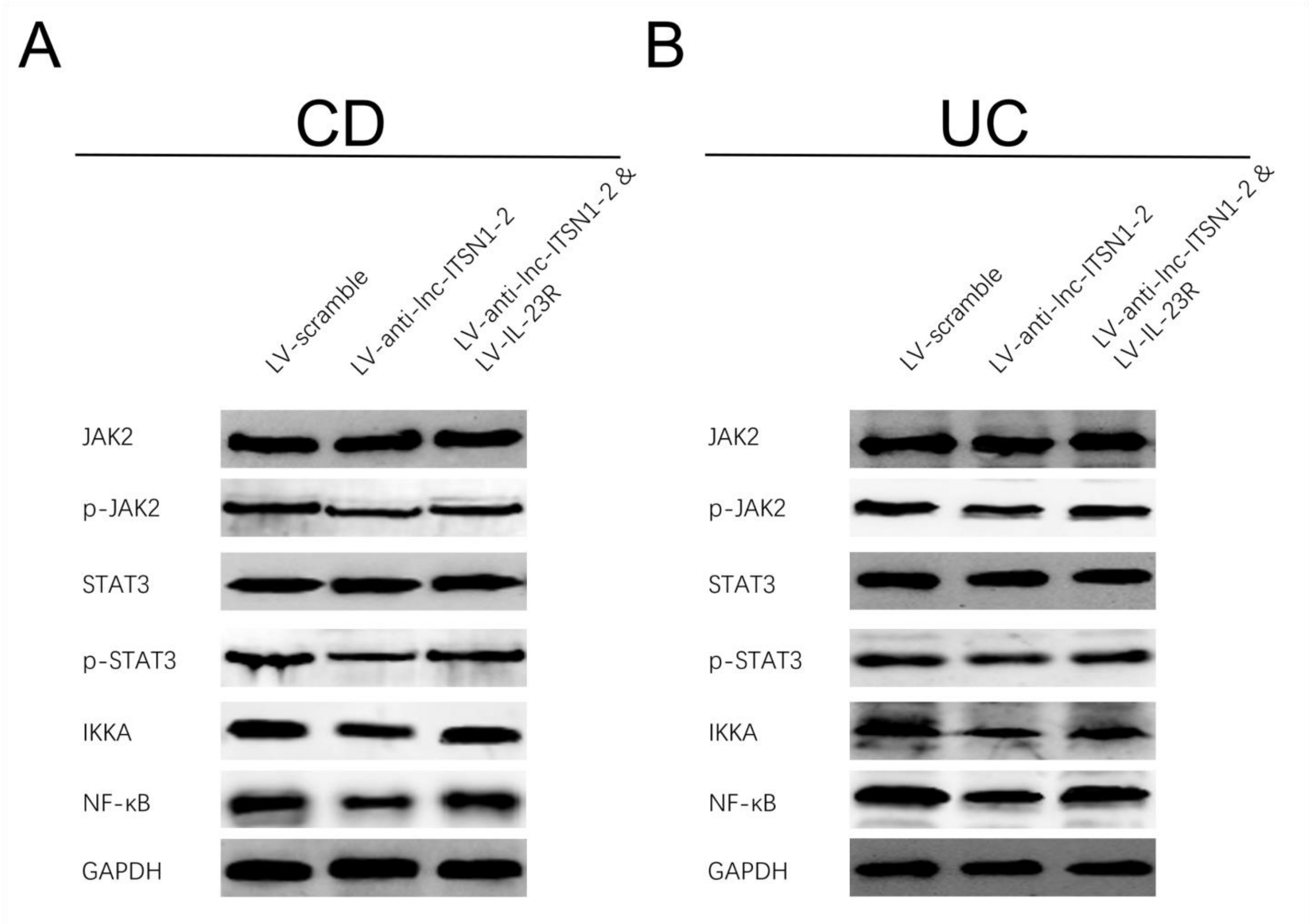

Supplementary figure 4. JAK2/STAT3 pathway and NF-κB pathway detection

JAK2, pJAK2, STAT3, pSTAT3, IKKA and NF-κB expressions in LV-scramble group, LV-anti-lnc-ITSN1-2 group, and LV-anti-lnc-ITSN1-2 & LV-IL-23R group of CD (A) and UC (B) $CD4^+$ T cells. CD, Crohn's disease; UC, ulcerative colitis.

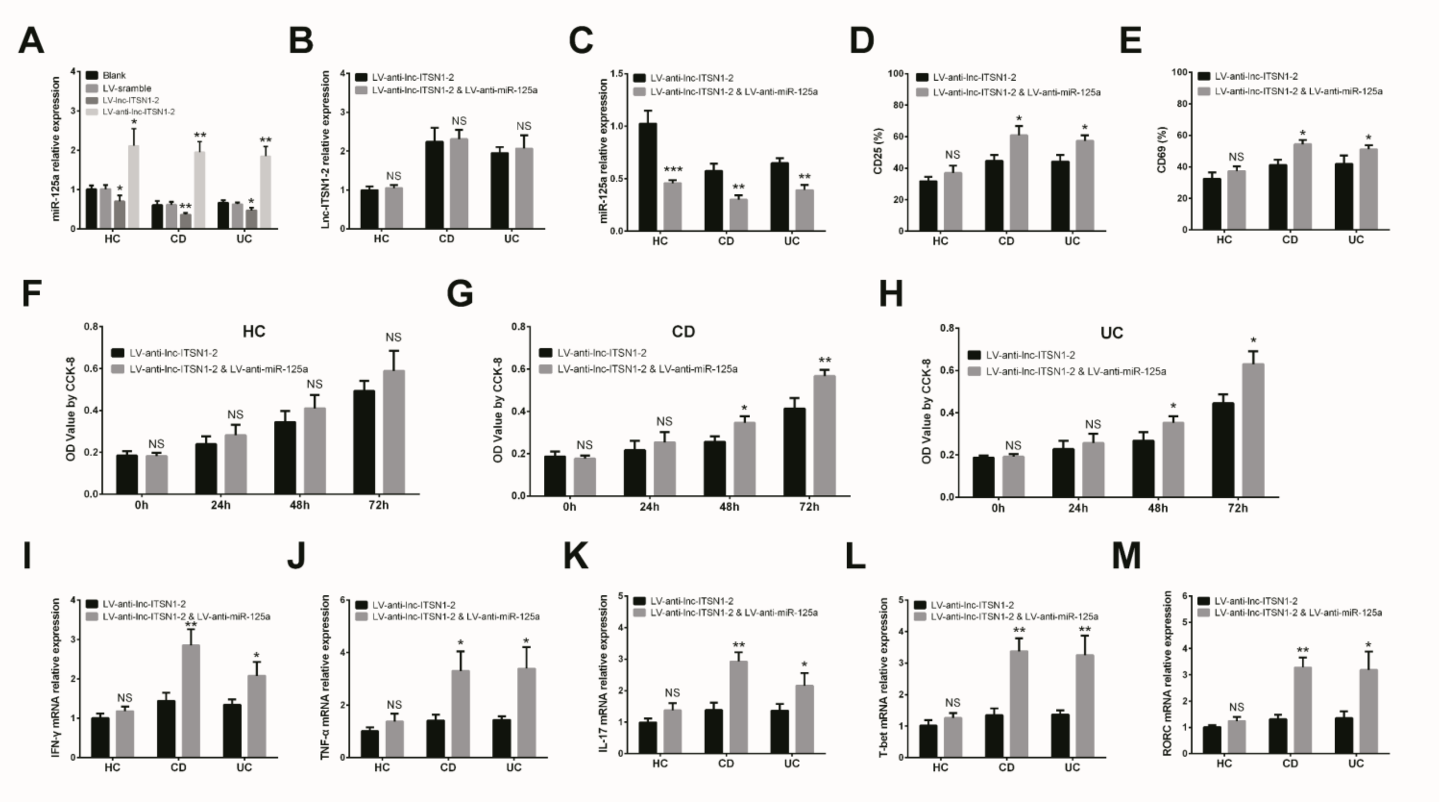

Supplementary figure 5. Rescue experiment: miR-125a knockdown in lnc-ITSN1-2 knockdown treated $CD4^+$ T cells. Comparison of miR-125a among groups (A). Comparison of lnc-ITSN1-2 expression (B), miR-125a expression (C), $CD25^+$ cell percentage (D), $CD69^+$ cell percentage (E), $CD4^+$ T cell proliferation (F-H), IFN-γ expression (I), TNF-α expression (J), IL-17 expression (K), T-bet expression (L), RORC expression (M) between LV-anti-lnc-ITSN1-2 & LV-anti-miR-125a group and LV-anti-lnc-ITSN1-2 group. NS, P>0.05; *, P<0.05; **, P<0.01; ***, P<0.001. HC, health control; CD, Crohn’s disease; UC, ulcerative colitis.

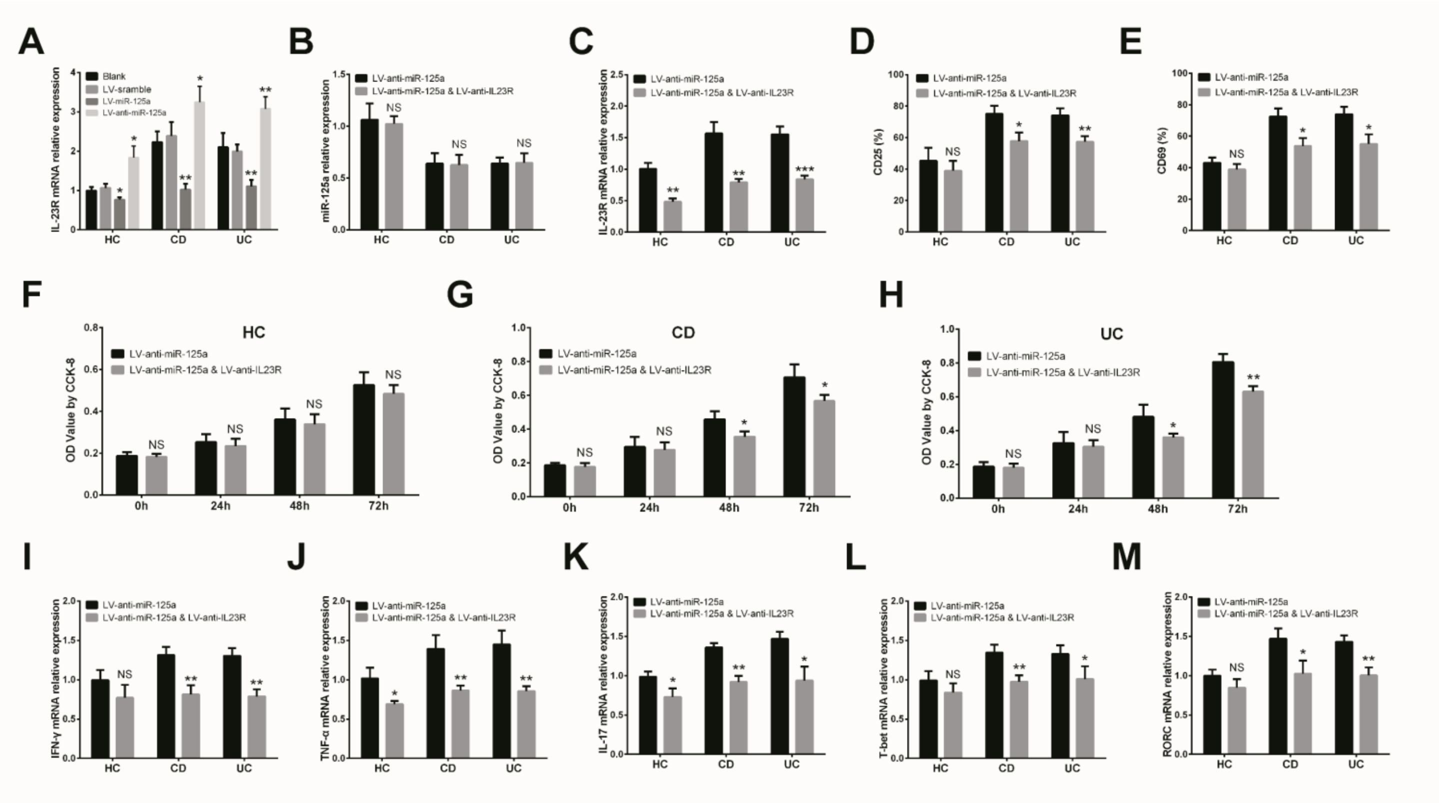

Supplementary figure 6. Rescue experiment: IL-23R knockdown in miR-125a knockdown treated $CD4^+$ T cells. Comparison of IL-23R among groups (A). Comparison of miR-125a expression (B), IL-23R expression (C), $CD25^+$ cell percentage (D), $CD69^+$ cell percentage (E), $CD4^+$ T cell proliferation (F-H), IFN-γ expression (I), TNF-α expression (J), IL-17 expression (K), T-bet expression (L), RORC expression (M) between LV-anti-miR-125a & LV-anti-IL23R group and LV-anti-miR-125a group. NS, $P>0.05$; *, $P<0.05$; **, $P<0.01$; ***, $P<0.001$. HC, health control; CD, Crohn's disease; UC, ulcerative colitis.

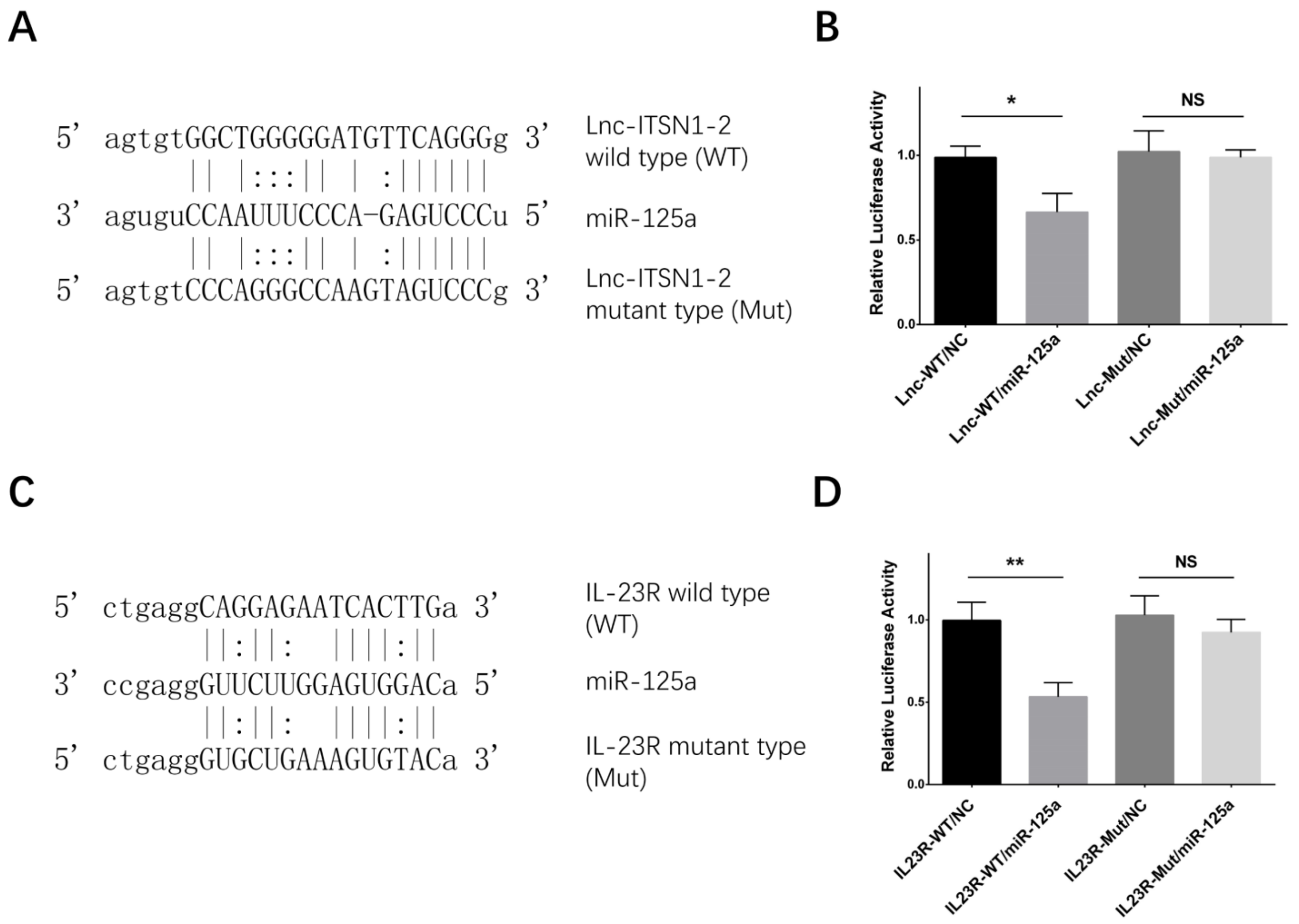

Supplementary figure 7. Luciferase Reporter assays. Designed mutant (Mut) sequence of lnc-ITSN1-2 binding to miR-125a (A), Relative luciferase activity among groups (B); Designed mutant (Mut) sequence of IL-23R binding to miR-125a (C), Relative luciferase activity among groups (D). NS, P>0.05; *, P<0.05; **, P<0.01.
